# AMPK modulates a DEAH box RNA-helicase to attenuate TOR signaling and establish developmental quiescence in *Caenorhabditis elegans*

**DOI:** 10.1371/journal.pbio.3003144

**Published:** 2025-12-01

**Authors:** Sabih Rashid, Richard Roy

**Affiliations:** Department of Biology, McGill University, Montreal, Canada; Institute of Molecular Biology, GERMANY

## Abstract

Developmental plasticity allows organisms to adapt to environmental stress and improve reproductive fitness. *Caenorhabditis elegans* adapts to starvation and other stressors by transiting through an alternate developmental stage called dauer, which allows them to remain quiescent for several months, and yet fully retain reproductive fitness when they resume development. The AMP-activated protein kinase (AMPK) is essential for animals to passage through the dauer stage without reproductive consequence. The loss of AMPK leads to germline hyperplasia, dramatically reduced post-dauer fertility, and shortened dauer survival. We identified a putative RNA-binding helicase (HZL-1) that is targeted by AMPK. Disabling HZL-1 function rescues many dauer and post-dauer reproductive defects typical of the AMPK mutants. HZL-1 shares significant similarity with the conserved HELZ family of RNA helicases, possessing characteristic DEAH helicase motifs, a predicted ATP binding motif, and intrinsically disordered regions that are crucial for its localization and function. Curiously, HZL-1 is expressed and exerts its function in the intestine, yet its elimination suppresses the aberrant germ cell proliferation while restoring germline quiescence and subsequent post-dauer fertility in AMPK mutants. CLIP-seq data revealed that HZL-1 binds several mRNAs during the dauer stage, and thus when it is active in AMPK mutants, its regulation of these RNAs contributes to germline hyperplasia in the dauer germ line. The most enriched RNA bound and inhibited by HZL-1, *argk-1*, promotes fertility by suppressing TOR activity in the germ line of dauer larvae, thereby preserving germline quiescence. These findings underscore the intricate role of RNAs and RNA-binding helicases in the complex interplay of genetic signals that animals have acquired to ensure their effective transit through periods of environmental challenge.

## Abstract 2

*Caenorhabditis elegans* adapts to starvation and other stressors by transiting through the quiescent developmental stage dauer, but the underlying mechanisms remain unclear. This study shows that TOR activity is inhibited by AMPK and a novel RNA-binding helicase in the germ line during dauer in order to preserve developmental quiescence.

## Introduction

Environmental fluctuations pose important challenges to all organisms, often limiting access to resources required for growth and reproduction. Through adaptation to these changes, some species have evolved means of altering their developmental trajectory in response to environmental or physiological stressors [1]. This “developmental plasticity” often involves periods of punctuated cell cycle quiescence [2], and in some cases, the execution of alternative developmental stages [3,4]. The decisions to execute one course versus another are usually very tightly regulated by genetic pathways that respond to specific environmental and/or developmental cues. This ensures the optimal developmental trajectory that will maximize fitness is robustly selected. One of the best-studied models for developmental plasticity is the nematode *Caenorhabditis elegans*, which exhibits numerous forms of plasticity, from the L1 larval stage diapause to the distinct dauer diapause, and even an ability to undergo arrest as reproductive adults [5–9].

The dauer diapause is of particular interest since many of the genetic mutations that alter this developmental decision are also involved in tissue growth, scaling, and longevity [10–12]. *C. elegans* larvae adopt this alternative developmental stage in response to suboptimal environmental conditions, including lack of nutrients, elevated temperature, and high population density. Transition into this stage permits the animals to survive these challenges until growth conditions improve [13]. These larvae are characterized by global developmental quiescence, altered metabolism, a modified protective cuticle, and widespread changes in gene expression [14]. Moreover, these developmental changes allow the animals to survive for up to four months without feeding, while the average life span of a nondauer *C. elegans* is approximately two to three weeks.

Remarkably, dauer larvae can quickly readjust their metabolism and resume their growth when environmental conditions improve, allowing them to return to a reproductive developmental trajectory. Furthermore, there are no obvious negative repercussions to fitness by passaging through the dauer stage, as animals remain fertile and have similar longevity to counterparts that do not enter the diapause state. However, post-dauer *C. elegans* do have an altered life history that is recorded in their chromatin, like a molecular memory, as well as slight changes in their reproductive capabilities, both of which persist transgenerationally [15,16].

A large number of signaling pathways must work in concert during the dauer stage, not only to induce the changes required for the transition into and out of dauer, but also to preserve the integrity of *C. elegans* cellular functions and ensure animals are fit and fertile following recovery from the dauer stage. Cellular pathways that otherwise promote cell proliferation and growth must respond to changes associated with dauer onset [17]. The signaling pathways that drive reproductive growth are attenuated, while the change in energy levels that is associated with the diapause state results in the activation of AMP-activated protein kinase (AMPK). Conversely, the target of rapamycin (TOR) signaling pathway has also been implicated in the formation of dauer larvae, as loss of TOR components results in the induction of dauer-like arrest, with corresponding changes in metabolism [18]. During the dauer stage, when nutrients are less abundant, TOR acts as a key signaling molecule that instructs the *C. elegans* larva to either adopt a reproductive mode of development, or to execute the diapause and to forego development until environmental conditions improve.

AMPK plays a significant role in establishing and maintaining germline quiescence during dauer. Previously, it was observed that loss of AMPK leads to germline hyperplasia during the dauer stage. Importantly, in addition to the appearance of various somatic defects in the post-dauer animals, most of the dauer larvae that do recover become sterile [19]. This is associated with a general misregulation of germline chromatin modifications, which has a significant impact on the gene expression of these animals, and may indeed contribute to the phenotypic changes characteristic of AMPK mutant dauer and post-dauer larvae.

Curiously, disabling the activity of some of the critical genes involved in the RNA interference pathway partially suppressed the germline hyperplasia and post-dauer sterility [19]. Therefore, AMPK must be involved in regulating appropriate small RNA homeostasis, as animals lacking AMPK have misregulated small RNAs in the dauer stage, suggesting they play a key role in regulating germline quiescence. However, the precise mechanisms by which AMPK influences RNA levels or function in order to preserve germline integrity are presently unclear.

Here, we show that a previously uncharacterized RNA-binding protein acts as a downstream target of AMPK that binds and inhibits RNAs that are critical for germline integrity during the dauer stage. Structural analysis suggests that this protein is related to DEAD/DEAH-box RNA helicases found in other species, such as HELZ [20,21], and as such we have named it HELZ-like 1, or HZL-1. Like many other members of this protein family, HZL-1 possesses intrinsically disordered regions (IDRs) that contribute to its function. As a putative phosphorylation target of AMPK, this protein functions in the intestine to regulate several target mRNAs, most notably the arginine kinase *argk-1*, ultimately impinging on the TOR signaling pathway. In the absence of AMPK signaling, HZL-1 binds to and inhibits the expression of its target RNAs, thereby preventing the required quiescence in the germ line of dauer larvae and promoting a proliferative signal through the activation of TOR. Our findings highlight a role for RNA-binding proteins in enabling the adjustment to nutrient stress by regulating the stability of specific mRNAs.

## Results

### A candidate RNAi survey to identify regulators of post-dauer fertility downstream of AMPK

Previous analyses of AMPK-deficient animals revealed defects in dauer germline quiescence and post-dauer fertility, some of which were suppressed by compromising specific RNA interference pathway components [19]. This suggests that the loss of AMPK resulted in the misregulation of small RNAs, although it was unclear how AMPK could impact small RNA levels, and how their putative target mRNAs could affect quiescence.

To observe dauer and post-dauer phenotypes, we used the temperature-sensitive *daf-2 (e1370)* strain, which constitutively forms dauer larvae when grown at the restrictive temperature of 25°C, but recovers into reproductive post-dauer animals when shifted back to 15 °C. Hereafter, all of our strains possess the *daf-2* allele to form dauer larvae at 25 °C, even if not otherwise indicated. For our AMPK mutants, we use strains harboring both *aak-1(tm1944)* and *aak-2(ok524)* mutations (hereafter referred to as “*aak(0)*”), which are loss-of-function (null) mutants of the two catalytic subunits that constitute AMPK.

To identify potential downstream targets of AMPK that regulate RNAs, we performed a bioinformatic analysis of the *C. elegans* protein sequences that possessed consensus AMPK phosphoacceptor sites [22], which might indicate they are direct targets of AMPK (listed in S2 Data). We further narrowed the list of targets by focusing on proteins predicted to be associated with small RNAs [23,24]. To then determine whether any of these targets played a functional role in regulating dauer germline quiescence, we performed a post-dauer fertility assay following RNAi against each of the predicted target genes, reasoning that a suppression of the post-dauer sterility seen in AMPK mutants could indicate the target was abnormally active in AMPK-deficient animals and contributed to the reproductive defect. This screen identified two targets that, when knocked down, resulted in a suppression of post-dauer fertility in AMPK animals: *parp-2*, a poly-ADP ribose polymerase, and an uncharacterized gene, C44H9.4 (S1A Fig).

**Fig 1:**
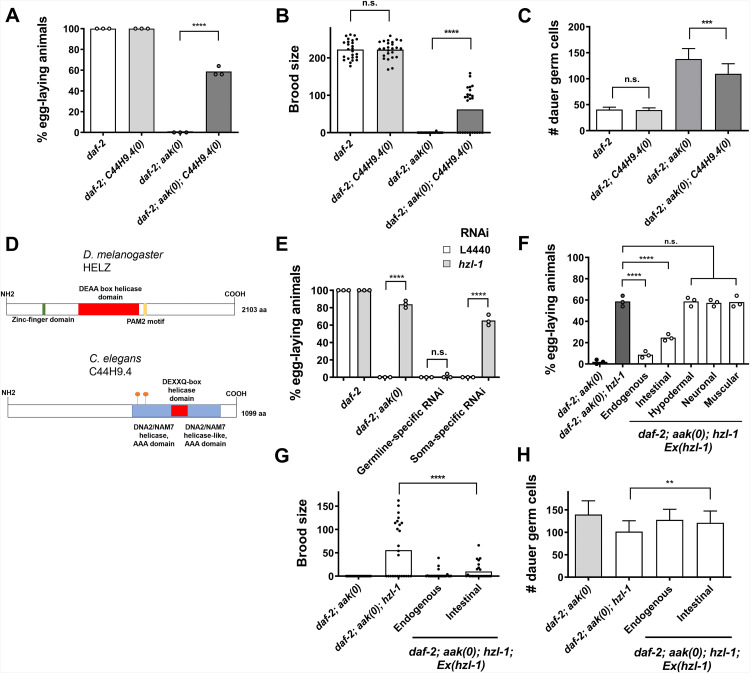
Disruption of C44H9.4/*hzl-1*, a putative RNA-binding helicase, suppresses post-dauer sterility in *daf-2; aak(0)* mutants. **A)** Quantification of post-dauer fertility of *daf-2*, **aak(0)*,* and C44H9.4/*hzl-1* mutants. **B)** Quantification of post-dauer brood size of *daf-2*, **aak(0)*,* and C44H9.4/*hzl-1* mutants. **C)** Quantification of germ cell count in *daf-2*, **aak(0)*,* and C44H9.4/*hzl-1* mutant dauer larvae. **D)** Schematic diagram depicting (top) the protein HELZ, a *Drosophila melanogaster* homolog of C44H9.4 based on BLASTP analysis and (bottom) *C. elegans* C44H9.4. Depicted RNA helicase domains predicted by InterPro. Putative AMPK phosphorylation sites at S588 and S636, as predicted by GPS 6.0 software, are shown as orange beacons. **E)** Quantification of post-dauer fertility in animals following tissue-specific RNAi of C44H9.4/*hzl-1* in *daf-2; aak(0)* animals. Whole animal RNAi (*daf-2; aak(0)*), soma-specific RNAi (*daf-2; aak(0); rde-1; sur-5p::rde-1*), and germline-specific RNAi (*daf-2; aak(0); rde-1; sun-1p::rde-1*) was performed. **F)** Quantification of post-dauer fertility in *hzl-1* mutant animals rescued by a wild-type copy of *hzl-1*. Transgenic insertion of a wild-type copy of *hzl-1* was performed in these mutants under the control of its endogenous promoter, or driven by an intestinal (*nhx-2*), hypodermal (*wrt-2*), neuronal (*rgef-1*), or muscular (*myo-3*) tissue-specific promoters. **G)** Quantification of post-dauer brood size of *daf-2*, **aak(0)*,* and *hzl-1* mutants rescued by wild-type *hzl-1* under the control of an endogenous or intestinal promoter. **H)** Germ cell count of *daf-2*, *aak(0)* and *hzl-1* mutants rescued by wild-type *hzl-1* under the control an endogenous or intestinal promoter. All post-dauer fertility data represent three independent trials, with the mean represented by columns and individual values by small circles. *n* = 50 for each trial. ****p < 0.0001 using one-way ANOVA for the indicated comparisons. All brood size assays and germ cell counts represent data from 25 individual animals per sample, with bars representing the mean and small circles representing individual values. ****p < 0.0001, ***p < 0.001, **p < 0.01 using one-way ANOVA for the indicated comparisons. The raw data underlying all figures can be found in S1 Data.

A genetic deletion mutant of C44H9.4, *ok1688,* displayed a similar suppression of post-dauer sterility (Fig 1A). However, even with this partial rescue, it was noted that the fertile animals had reduced brood size compared to healthy *daf-2* controls (Fig 1B). We carried out DAPI staining of dauer larvae to determine whether the loss of C44H9.4 impacted the germ cell numbers, and observed a partial reduction of germline hyperplasia in these mutants (Figs 1C and S1B). Loss of C44H9.4 thus suppresses some of reproductive defects of *aak(0)* mutants, but does not fully restore the germ line to the healthy state of *daf-2* control animals.

Conversely, RNAi against *parp-2* in this background did not result in additive effects on fertility (S1C Fig), suggesting the two genes may be acting in a common linear pathway. Loss of either gene in control dauer backgrounds (*daf-2)* had no effect on fertility, nor did it cause any observable phenotypes. We chose to focus on C44H9.4 for further study due to its novel nature, as well as its putative roles in RNA regulation.

Structural predictions of the C44H9.4 protein indicate that it contains DNA2/NAM7 helicase domains, as well as a DEXXQ-box helicase domain (Fig 1D). Furthermore, sequence comparisons indicated the predicted C44H9.4 protein is a homolog of an RNA-binding helicase called HELZ, which has previously been characterized in *Homo sapiens* and *Drosophila melanogaster* [20,21]. Notably, C44H9.4 lacks the Zinc-finger domain and PAM2 motif present in HELZ, suggesting that its function may have diverged, despite having retained the RNA-binding motifs. Nevertheless, due to the shared similarities between these proteins, we will hereafter refer to the protein encoded by C44H9.4 as HELZ-like 1, HZL-1 or *hzl-1* (gene/transcript).

Given that the loss of *hzl-1* was sufficient to restore fertility in post-dauer AMPK mutants, we questioned whether its function was required autonomously in the germ line per se, or if it acted non-cell autonomously similar to AMPK [19]. Tissue-specific RNAi performed against *hzl-1* in the germ line did not restore fertility, whereas soma-specific RNAi was sufficient to suppress sterility nearly to the same levels as whole-animal *hzl-1* RNAi (Fig 1E), hinting that the protein may function in tissues other than the germ cells, but communicates information to the germ line.

To identify in which tissues *hzl-1* may be active, we inserted a transgenic wild-type copy of the protein into the mutant strain under its endogenous promoter, as well as various tissue-specific promoters. Expression of *hzl-1* under its own endogenous promoter led to near-complete reversion of the suppression of post-dauer sterility, as animals were observed to be mostly sterile, along with a greatly reduced brood size and increased germ cell count, suggesting that *hzl-1* gene function was restored (Fig 1F–1H). In addition, intestinally-expressed *hzl-1* also partially reverted the suppression, with significantly more of these transgenic animals being post-dauer sterile compared to the mutant strain, whereas expression under other tissue-specific promoters had no effect. These data therefore suggest that HZL-1 functions in the intestine of AMPK mutant larvae, and is sufficient to mediate its effects on germline quiescence during the dauer stage.

The domain predictions for HZL-1 suggest it may be an RNA-binding helicase. Specifically, it is predicted to contain a DEXXQ box helicase domain, as well as DNA/NAM2 domains (Fig 1D). Given that the protein influences the germ line and reproductive capability, we wondered whether it was similar to other helicases with related functions. For example, ZGRF1 is a helicase in mammalian models that promotes DNA repair through homologous recombination, and has functions in meiotic recombination [25,26]. Both HZL-1 and ZGRF-1 share similar helicase domains, although ZGRF-1 possesses a zinc-finger domain that HZL-1 lacks. Other helicases such as DNA-2 in *C. elegans* are critical for embryonic viability through their maintenance of genome integrity [27]. Some helicases also bind RNA, including small RNA, in order to preserve germline function. These include ZNFX-1, a major component in Z granules [28], and ERI-6/7, both RNA helicases required for biogenesis of ERGO-1 class 26G small RNAs which in turn regulate gene expression in the oocyte [29,30].

Given the similarities between HZL-1 and other helicases such as ZGRF1, we wondered whether HZL-1 was also playing a role in meiotic recombination or related functions. The loss of meiotic regulation often manifests as dead embryos or an increased incidence of male progeny [31]. We observed no instances of dead embryos in *hzl-1* single mutants, nor in any other genetic background during development. We measured the frequency of males in these strains by separating individual hermaphrodites and allowing them to lay eggs, to then score the percentage of males. Regardless of genetic background, we did not observe any changes in the percentage of males in *hzl-1* mutant progeny (S1D Fig). Our findings suggest that HZL-1 may not impact the germ line through regulation of meiotic recombination, as other related RNA helicases do. However, given the presence of a DEXXQ domain in its protein sequence, it may nevertheless interact with RNA, either mRNA or small RNAs, in order to influence reproductive functions.

### A putative AMPK phosphorylation site within HZL-1 is required for its function

**Fig 2:**
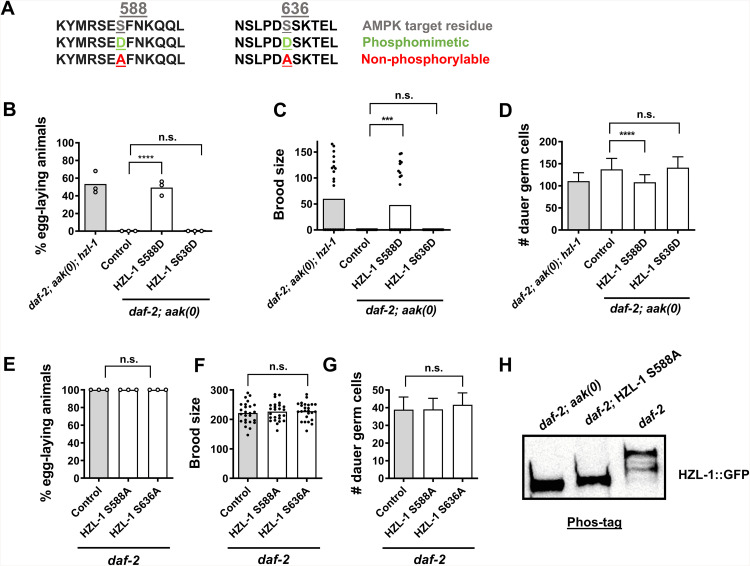
A putative AMPK phosphorylation site on HZL-1 modulates its function. **A)** Amino acid sequence of the predicted AMPK phosphorylation site at serine 588 or serine 636 in HZL-1 including indicated phosphomimetic/phosphonull (non-phosphorylable) changes (**D** and **A** in green and red, respectively). **B–D)** Post-dauer fertility, brood size, and germ cell counts of *daf-2; aak(0)* animals with phosphomimetic point mutations in *hzl-1*. **E–G)** Post-dauer fertility, brood size, and germ cell counts of *daf-2* animals with non-phosphorylable variant point mutations in *hzl-1*. All post-dauer fertility data represent three independent trials, with the mean represented by columns and individual values by small circles. *n* = 50 for each trial. ****p < 0.0001, using one-way ANOVA for the indicated comparisons. All brood size assays and germ cell counts represent data from 25 individual animals, with bars representing the mean and small circles representing individual values. ****p < 0.0001, ***p < 0.001 using one-way ANOVA for the indicated comparisons. **H)** Representative Phos-tag western blot depicting relative migration of HZL-1::GFP bands in indicated mutant strains, as detected by anti-GFP antibodies. *daf-2; aak(0)* mutant samples are compared with *daf-2* controls as well as *daf-2; hzl-1* mutants rescued with the S588A *hzl-1* variant. All strains possess the *hzl-1* mutation rescued by transgenic insertion of *hzl-1* under the intestinal *nhx-2* promoter. Western analyses were performed on Day 2 dauer larvae for each experiment. Approximately ~600 dauer larvae were used for each well, run on a 6% SDS-PAGE Phos-tag gel for 45–90 min, as needed. The raw data underlying all figures can be found in S1 Data. Original blots can be found in S1 Raw Images.

Loss of HZL-1 in AMPK-deficient animals corrects several defects, suggesting the helicase may be aberrantly active in AMPK mutants. We thus hypothesized that it is inhibited by AMPK, likely through phosphorylation. HZL-1 has two predicted AMPK phosphorylation motifs in its amino acid sequence. Phosphorylation by AMPK can result in the inhibition of proteins through various means, including the induction of 14-3-3 binding to the target [32]. To determine whether AMPK regulates HZL-1 activity by phosphorylation, we mutated the predicted phosphoacceptor sites on the protein to generate variants that were phosphomimetic, by replacing the acceptor serine with an aspartic acid, or non-phosphorylable by replacing the same serine residue with a neutral alanine (Fig 2A).

Initially both phosphovariant strains were prepared by transgenically expressing an altered copy of *hzl-1,* rescuing *hzl-1* mutants in either *daf-2* or *aak(0)* backgrounds (S2 Fig). Subsequently, we generated strains via CRISPR-mediated mutation within the endogenous *hzl-1* locus (Fig 2). The phosphomimetic variant of HZL-1 was generated in AMPK mutant strains, and we reasoned that the phosphomimetic variant would mimic phosphorylation by AMPK, where HZL-1 activity would presumably be attenuated and thus function similarly to the deletion mutant of HZL-1, resulting in fertile post-dauer AMPK mutant adults. Conversely, a non-phosphorylable variant generated in a *daf-2* control background might result in HZL-1 being aberrantly active, since such a protein would no longer be regulated by AMPK, and thus might escape from germline quiescence in the dauer stage, despite the presence of active AMPK.

When assessing the post-dauer fertility of these variants, we noted that the phosphomimetic S588D strain was indeed post-dauer fertile, and had reduced germline hyperplasia, despite it lacking all AMPK signaling, very similar to AMPK mutant animals that lacked *hzl-1* (Figs 2B–2D and S2A). This suggests that AMPK could potentially phosphorylate HZL-1 at S588, thereby disabling it. However, the independent mutation of the second site at S636 appeared to have no effect, as transgenic animals harboring this phospho-site variant exhibited similar levels of fertility to the intact HZL-1 transgene control. We did not observe an additive effect of combining the two mutated sites, suggesting that S588 is the only phosphorylable residue that is functionally relevant in this context.

When assessing the fertility of the non-phosphorylable variants in the *daf-2* background, we observed no changes in fertility, as all animals were fully fertile similar to the control (Figs 2E–2G and S2B). Furthermore, mutation of both sites had no effect on any of the observed phenotypes. This indicates that even if HZL-1 is active during the dauer stage due to a lack of phosphorylation by AMPK, its active state may not be sufficient alone to interfere with germline integrity. Alternatively, other targets that act downstream of AMPK signaling likely compensate for the non-phosphorylable HZL-1 variant, either by inhibiting the protein itself, or by promoting a parallel pro-quiescent signal that circumvents any effect of HZL-1 [33].

To confirm that phosphorylation of HZL-1 occurs at the S588 site, we performed Western analysis using Phos-tag gels.

These modified SDS-PAGE gels incorporate Phos-tag acrylamide to allow for the detection of phosphorylation events via the binding of metal ions within the gel to phosphate monoesters. Phosphorylated proteins bound by these metal ions migrate slower on the gel, thus distinguishing them from non-phosphorylated proteins [34]. We assayed the phosphorylation state of HZL-1 from *aak(0)* dauer larvae using a GFP-tagged HZL-1, and compared the migration of the GFP band with samples from *daf-2* dauer larvae (AMPK+), where HZL-1 is presumably phosphorylated. We also tested our S588A non-phosphorylable variant strain to determine if this site was potentially phosphorylated by AMPK. We observed the HZL-1::GFP band in the *daf-2* sample ran visibly higher than the HZL-1::GFP expressed in the *aak(0)* animals, suggesting that the protein is indeed phosphorylated when AMPK is active (Fig 2H). Consistent with AMPK targeting HZL-1 at S588, the S588A HZL-1::GFP variant in the *daf-2* background migrated at a rate similar to the HZL-1::GFP expressed in the AMPK mutants. These data support the conclusion that HZL-1 is phosphorylated at S588 by AMPK during the dauer stage.

If HZL-1 is indeed an RNA-binding helicase, differences in structure induced via phosphorylation may impact the ability of the protein to bind RNA, as both the electrostatic charge as well as physical structure impacts interactions between RNA and protein [35,36]. We looked at the predicted structures of both the wild-type and the S588D phosphomimetic variant of HZL-1 using AlphaFold [37]. There is no obvious difference in the overall structure of the two proteins when compared side by side, except for a small increase in the spacing around the residue (S2C and S2D Fig). With this limited structural information it is difficult to definitively state whether phosphorylation could change protein structure sufficiently to influence its RNA-binding properties. More detailed analyses would be required to more accurately determine how phosphorylation impacts HZL-1 function.

Phosphorylation by AMPK can affect protein function in other ways, including degradation of the protein or sequestering to different cellular compartments [32,38,39]. Many AMPK targets are sequestered and degraded through generation of 14-3-3 target sites following phosphorylation [32,40]. To identify whether phosphorylation by AMPK results in the destabilization of HZL-1, we verified the levels of the HZL-1 phosphomimetic variants using a GFP-tagged HZL-1. Our observations via western blot indicated that the protein levels of our phosphomimetic variants remained largely unchanged compared to the wild type or the non-phosphorylable variants, suggesting that AMPK-mediated phosphorylation modulates HZL-1 function through some other means rather than enhancing their degradation (S2E and S2F Fig).

We then wanted to know whether the cellular localization of the protein might be affected by phosphorylation. It is difficult to image HZL-1 due to the low endogenous levels of expression, as we confirmed with our HZL-1::GFP transgenic strain expressed under the endogenous *hzl-1* promoter. We therefore used the intestinally-expressed variant, which we showed can restore fertility in the post-dauer AMPK mutants, suggesting that both the tissue-specific expression and the expression levels are compatible with HZL-1 function (Fig 1D). HZL-1::GFP is strongly expressed in the intestine (S2G Fig). Furthermore, when we express the S588 phosphomimetic under the intestinal promoter, we see the same pattern and intensity of expression, suggesting that the additional charge conferred by the aspartate in the phosphomimetic variant does not result in any change in the cellular localization, or the abundance of HZL-1. The AMPK-mediated attenuation of HZL-1 function must therefore rely on some other phosphorylation-associated modulation via an alternate mechanism, such as a conformational change [33].

### Intrinsically disordered regions in HZL-1 are required for its expression during dauer and its regulation of post-dauer fertility

**Fig 3:**
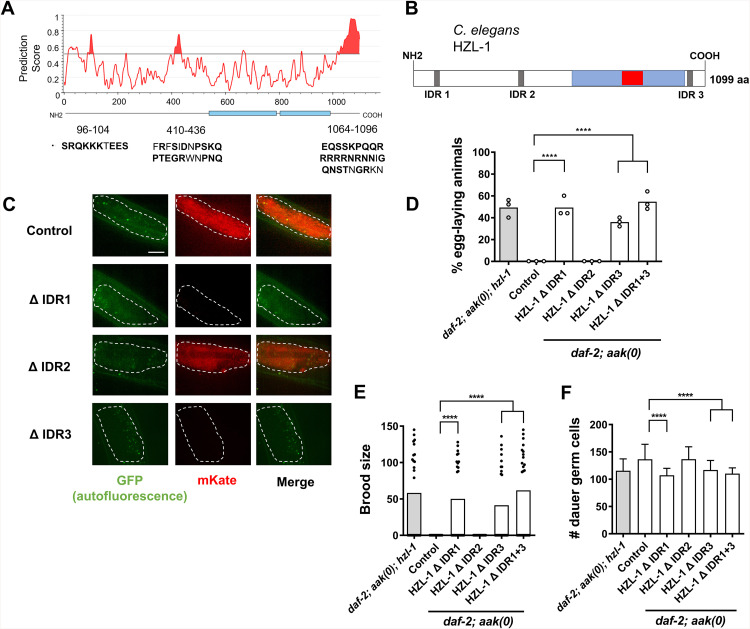
HZL-1 possesses three predicted IDRs that are differentially required for its expression and function. **A)** Intrinsically disordered domains present in the HZL-1 amino acid sequence as predicted by IUPred3. Residues that correspond to the predicted peaks are indicated below the graph in capital letters, with disorder-contributing residues in bold. **B)** Updated protein schematic of HZL-1 with the location of predicted IDRs indicated by gray rectangles. **C)** Confocal images of HZL-1 expressed under the intestinal promoter (*nhx-2*) in *aak(0); hzl-1* mutants. Dauer larvae (Control) were all *daf-2* maintained at 25 °C. Each micrograph shows the posterior intestine that expresses transgenic HZL-1 variants with deletions of individual IDR sequences (IDR1, IDR2 and IDR3). Scale bar = 10 µm. **D–F)** Post-dauer fertility, brood size and germ cell counts of *daf-2; aak (0)* animals with various IDR deletions in *hzl-1*. All post-dauer fertility data represent three independent trials, with the mean represented by columns and individual values by small circles. *n* = 50 for each trial. ****p < 0.0001, using one-way ANOVA for the indicated comparisons. All brood size assays and germ cell counts represent data from 25 individual animals per sample, with bars representing the mean and small circles representing individual values. ****p < 0.0001, using one-way ANOVA for the indicated comparisons. The raw data underlying all figures can be found in S1 Data.

Many RNA-binding helicases are able to form liquid-liquid condensates that facilitate further interactions with RNAs or other proteins [41]. Intrinsically disordered regions (IDRs) within the protein can confer the ability to form these condensates and are frequently observed in RNA helicases [42,43]. Given the predicted domains on HZL-1 indicate that it could be an RNA-binding helicase, we wondered whether the protein also harbored IDRs, and whether they might contribute to its function. Analysis of the amino acid sequence using the resource IUPred3 [44] revealed that HZL-1 possesses three predicted IDRs (Fig 3A and 3B).

To further investigate whether these IDRs contribute to the function of HZL-1, we designed a series of transgenic variants with single or compound deletions in one or more of the predicted IDRs and expressed them in the *hzl-1* null mutant, and additionally generated CRISPR deletions of the IDRs within the endogenous HZL-1 locus in AMPK mutants. Using an mKate-tagged HZL-1 expressed under an intestinal promoter, we detected strong fluorescent signal in the intestine of dauer larvae when we drove the expression of an intact wild-type HZL-1 protein (Fig 3C, top row). In contrast to the wild-type protein, the IDR1 or IDR3 deletions were undetectable during the dauer stage (Fig 3C, rows 2 and 4), whereas the IDR2 deletion had no effect, as fluorescence was similar to the wild-type control. To confirm that deletion of the IDRs did not affect the expression of *hzl-1*, we performed RT-qPCR to measure levels of *hzl-1* in our IDR deletion strains, and found that the levels of mRNA were consistent regardless of whether wild-type or IDR variants of *hzl-1* were expressed (S3A Fig).

Furthermore, analysis of the post-dauer fertility of the ΔIDR transgenic strains revealed that loss of IDR1 or 3 increased their fertility compared to the control transgenic strain expressing wild-type HZL-1 (Fig 3D). This suggests that these variants of the protein impede the function of HZL-1 such that unlike its wild-type counterpart, even though the protein is expressed, it does not disrupt germline quiescence because it is not functional. These findings are consistent with the observed changes in protein expression, further corroborating the requirement of IDR1 and IDR3 for the proper activity, or stability of HZL-1 during the dauer stage. Furthermore, the significance of IDRs within HZL-1 provides further evidence that it may be an RNA-binding helicase, as IDRs are often necessary for helicases that regulate RNAs [36].

Furthermore, analysis of the post-dauer fertility of the ΔIDR transgenic strains revealed that loss of IDR1 or 3 increased their fertility compared to the control transgenic strain expressing wild-type HZL-1 (S3B Fig). This was confirmed by our CRISPR deletion strains, where we assessed post-dauer fertility, brood size, and germline hyperplasia (Fig 3D–3F). Similar to our transgenic strains, these mutants revealed HZL-1 function appeared to be impaired when IDR1 or IDR3 were removed, as animals had increased fertility and reduced germline hyperplasia. This indicates these IDR deletion variants of the protein impede the function of HZL-1 such that, unlike its wild-type counterpart, even though the protein is expressed, it does not disrupt germline quiescence because it is not functional. This is consistent with the observed changes in protein expression, further corroborating the requirement of IDR1 and IDR3 for the proper activity, or stability of HZL-1 during the dauer stage. Furthermore, the significance of the IDRs within HZL-1 provides further evidence that it may be an RNA-binding helicase, as IDRs are often present and required for RNA helicases to bind and regulate their target RNAs [45].

### Transcriptomic analysis of AMPK and HZL-1 mutants reveals widespread gene expression differences primarily in the germ line

The structure and the conserved domains of HZL-1 are consistent with it acting as an RNA-binding protein that may inappropriately affect the levels of growth- or proliferation-specific RNAs in dauer larvae when AMPK activity is compromised. To better understand what changes in RNA homeostasis may be induced by the protein, we performed a transcriptomic analysis of the *hzl-1* deletion mutant. We obtained total RNA from dauer and post-dauer animals that were of four different genetic backgrounds: *daf-2* (dauer control), *daf-2; aak(0)* (AMPK mutant), *daf-2; hzl-1* (HZL-1 mutant control), and *daf-2; aak(0); hzl-1* (HZL-1 and AMPK double mutant). These samples were then submitted for mRNA sequencing, and bioinformatic analysis was subsequently performed (S4A Fig).

**Fig 4:**
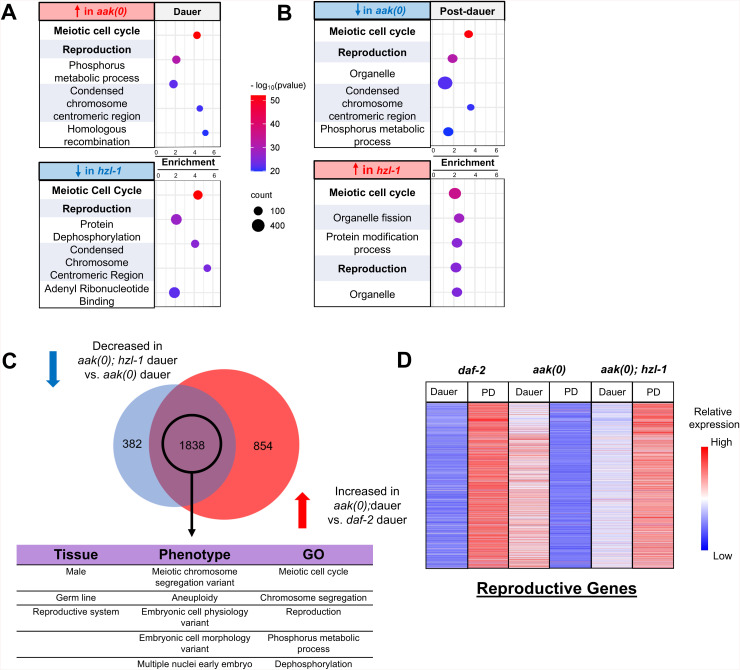
Transcriptomic analysis of AMPK and HZL-1 mutants reveals widespread gene expression differences predominantly in the germ line. **A)** and **B)** Bubble plots depicting most enriched GO terms in *daf-2*, **daf-2, aak(0)*,* and *aak(0); hzl-1* transcriptomes, ranked by significance based on p-value. Changes in gene expression in dauer larvae depicted in **A)**, and post-dauer in **B)** Top: Comparison of *aak(0)* vs. *daf-2* animals. Bottom: Comparison of *hzl-1; aak(0)* vs. *aak(0)* animals. Size of bubbles indicate number of genes in their respective categories that were enriched in the dataset. GO enrichment was conducted using the Wormbase Gene Set Enrichment Analysis [46,47]. **C)** (Top) Venn diagram comparing genes with decreased expression in *hzl-1; aak(0)* dauer vs. *aak(0)* dauer, and genes with increased expression in *daf-2; aak(0)* dauer vs. *daf-2* dauer, indicating a large overlap of 1838 genes. Bottom: Tissue, phenotype, and gene ontology enrichment of overlapped genes, highlighting an enrichment in genes associated with the germ line and reproductive phenotypes. **D)** Heat map of the expression of ~2,000 reproductive genes from **(C)**. This subset of genes is differentially expressed in *aak(0)* and *hzl-1; aak(0)* mutants, as well as in dauer and post-dauer animals. Data represents normalized transcripts per million values for each gene, with higher relative expression in red and lower in blue. The raw data underlying all figures can be found in S1 Data.

Transcriptional analysis of these mutants revealed widespread gene expression changes during both the dauer and post-dauer stages (S4B Fig). We noted a large number of differentially expressed genes when comparing *aak(0)* to control *daf-2* animals, and comparing *aak(0); hzl-1* animals to *aak(0)* samples. We then performed GO enrichment of our transcriptomic comparisons [46,47], and identified reproductive and germ line genes as being the most enriched in the gene set that had increased expression in *aak(0)* dauer animals compared to controls (Fig 4A). Notably, this pattern of expression was inverted in the post-dauer comparison (Figs 4B, S4C, and S4D), suggesting reproductive genes are expressed at relatively lower levels in AMPK mutant post-dauer adult animals, potentially because their reproductive developmental program was prematurely activated during the dauer stage. Loss of AMPK thus appears to induce reproductive development aberrantly, even in the dauer stage, where animals are expected to be quiescent. This correlates with our phenotypic analysis of these mutants, which reveals germline hyperplasia and premature spermatogenesis that occurs during the dauer stage [17].

We noted a similar inverse pattern of expression when comparing *aak(0); hzl-1* mutants to *aak(0)* (Figs 4A, S4C, and S4D). Specifically, the GO enrichment in the subset that was decreased in *hzl-1* mutant dauer animals is comparable to the subset that is increased in AMPK mutant dauer data set. A similar pattern is seen when observing the post-dauer datasets, where reproductive and germline genes have increased expression in *hzl-1* mutants compared to *aak(0)* (Figs 4B, S4C, and S4D). This suggests the loss of *hzl-1* reverses the transcriptomic changes induced by the absence of AMPK, consistent with our findings that mutation of *hzl-1* partially restores fertility to these animals.

We further compared the subset of genes with increased expression in *aak(0)* dauer versus *daf-2* control (Fig 4C, left) with those with decreased expression in the *aak(0); hzl-1* mutant versus *aak(0)* (Fig 4C, right) and identified a significant overlap. Enrichment analysis of this shared gene set suggests they are, indeed, primarily reproductive genes. When comparing expression of these reproductive genes across our datasets, we noted that the *aak(0)* dauer transcriptome is strikingly similar to that of *daf-2* control post-dauer, whereas the *aak(0); hzl-1* dauer transcriptome is closer to *daf-2* dauer (Fig 4D). This is true for the post-dauer gene expression of *aak(0); hzl-1* mutants as well. We conclude that HZL-1 likely affects an ensemble of transcripts that are involved in the induction of quiescence to ensure germ cell growth and cell proliferation during larval development, namely during the L3 stage. Under dauer-inducing conditions, its activity is attenuated by AMPK-mediated phosphorylation. However, in the *aak(0)* mutant background, HZL-1 is abnormally active in the dauer stage and promotes reproductive development through effects on the transcriptome, contributing to the defects seen in these mutants. As a result, in the *aak(0); hzl-1* mutant background, the transcriptome largely comprises mRNAs that are more typical of a replete state, at least as it pertains to reproductive and germline genes.

### HZL-1 binds mRNAs which may contribute to its regulation of dauer germline quiescence in AMPK mutants

We observed widespread changes in mRNA levels resulting from the loss of HZL-1, but it was unclear how this protein was able to have such a major effect on the *C. elegans* transcriptome. Since HZL-1 possesses all the structural features for it to act as an RNA-binding protein, we sought to verify if HZL-1 could indeed bind specific RNAs to modulate their levels, such that when it is misregulated these RNAs might perturb the ability of AMPK mutants to maintain dauer quiescence.

We performed cross-linking immunoprecipitation on HZL-1 followed by RNA sequence analysis (CLIP-seq), by using our intestinally-expressed GFP-tagged HZL-1 strain, which we previously demonstrated was sufficient to restore HZL-1 function (protocol in S5A Fig). Using both *daf-2* control and *daf-2; aak(0)* strains harboring this transgene, we used an anti-GFP antibody to immunoprecipitate RNA targets from a total RNA extract obtained from each genotype. The bound RNAs were then collected and were subjected to mRNA-seq and small RNA-seq followed by bioinformatic analysis. As a negative control, we also carried out the IP and RNA extraction on a strain expressing GFP under the same intestinal promoter, but without HZL-1.

The CLIP-seq experiment revealed that numerous RNAs were bound by HZL-1 in both the control (*daf-2)* and *aak(0)* mutant backgrounds. Notably, the small RNA sequencing revealed no small RNAs were bound to HZL-1, suggesting that HZL-1 binds primarily to mRNAs. We compared the data from the *daf-2* and *aak(0)* samples to the negative control (i.e., only GFP) in order to identify any bound targets that may simply be noise or background, and eliminated targets from analysis that were strongly detected in the negative control.

**Fig 5:**
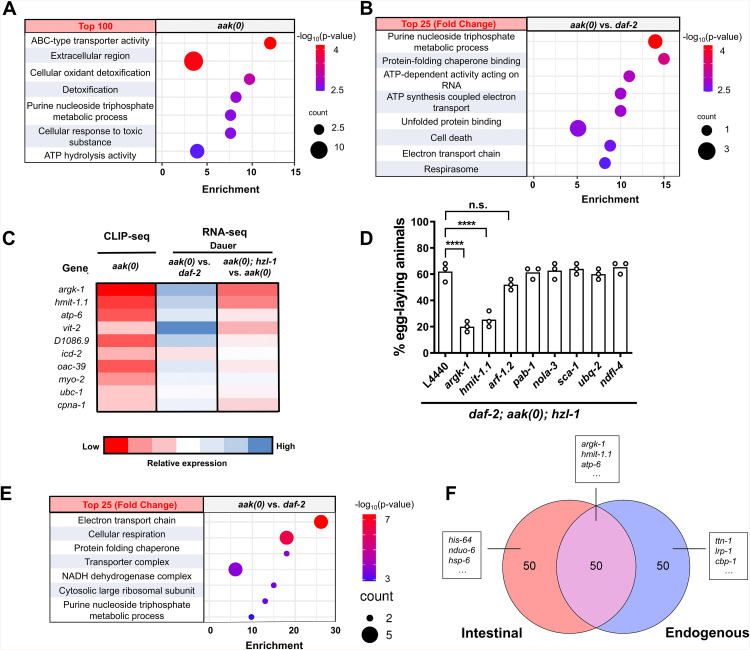
HZL-1 binds numerous RNAs and negatively regulates their expression. **A)** and **B)** Bubble plots depicting most enriched GO terms in RNAs bound to HZL-1, based on CLIP-seq data, ranked by significance based on p-value. **A)** Enrichment of top 100 RNAs bound to HZL-1 in *aak(0)* mutants. **B)** Top 25 RNAs with biggest fold change increase in the *aak(0)* dataset compared to *daf-2.* Size of bubbles indicate number of genes in their respective categories that were enriched in the indicated dataset. GO enrichment was conducted using the Wormbase Gene Set Enrichment Analysis [46,47]. **C)** Heat map of the most highly enriched genes in the *aak(0)* CLIP-seq dataset, compared to fold changes of those genes in the *aak(0)* dauer vs. *daf-2* dauer, and *hzl-1(0); aak(0)* dauer vs. *aak(0)* dauer RNA-seq datasets. Heat maps generated based on relative expression for CLIP-seq data, and log2 fold change for RNA-seq. **D)** Post-dauer fertility of *daf-2; aak(0); hzl-1(0)* animals following RNAi against select targets enriched in CLIP-seq *aak(0)* dataset. L4440 empty vector serves as a control. Post-dauer fertility data represent the results from three independent trials, where the mean is represented by columns and values for individual trials indicated by small circles. *n* = 50 for each trial. ****p < 0.0001 using one-way ANOVA for the indicated comparisons. **E)** Bubble plots depicting the most enriched GO terms in RNAs bound to HZL-1, expressed under its endogenous promoter, based on CLIP-seq data, and ranked by significance based on *p*-value. Enrichment depicts top 25 RNAs with biggest fold change increase in *daf-2; aak(0)* dataset compared to *daf-2*. **F)** Venn diagram comparing top 100 most enriched RNAs in CLIP-seq samples of intestinal vs. endogenously expressed HZL-1 in the *aak(0)* background. Boxes list several unique RNAs found in each subset. The raw data underlying all figures can be found in S1 Data.

We next sought to determine what types of RNAs were bound to HZL-1. Gene enrichment analysis of the bound RNAs revealed a diverse set of gene categories that were present in the CLIP (Fig 5A and 5B). We analyzed the most enriched RNAs in both the *daf-2* (S5B Fig) and *aak(0)* (Fig 5A) samples, as well as those that had a significant fold change increase when comparing the *aak(0)* sample to our *daf-2* controls (Fig 5B). In the *daf-2* background, we noted enrichment of RNAs associated with a wide variety of functions, including life span and development (S5B Fig). The most enriched targets in the *aak(0)* background were associated with metabolism and detoxification (Fig 5A), which may in part reflect our choice of promoter for HZL-1 expression, given it was intestinal. We saw no enrichment of germ line or reproduction-associated gene transcripts. This was curious as we showed that HZL-1 exerts its effects on the germ line of dauer animals, but it does not directly regulate mRNAs expressed from germ line-associated genes.

When looking at those RNAs with the biggest fold change difference between our *aak(0)* and *daf-2* samples, we saw an increase in metabolic target enrichment, specifically associated with the electron transport chain and related functions. This may reflect the role of HZL-1 in the intestine, a tissue that responds to diet and nutrition through adjustments to metabolism [48]. Furthermore, in the absence of the metabolic regulator AMPK, perhaps HZL-1 specifically targets RNAs related to energy metabolism. This regulation could potentially influence the metabolic phenotypes that we have previously characterized in AMPK mutant dauer larvae [49].

One notable target of HZL-1 that we obtained in our CLIP-seq data was *argk-1*, which was the most enriched RNA in our *aak(0)* samples across three biological replicates, and not present at all in the control. ARGK-1 is an arginine kinase that is similar to creatine kinases in mammals, and was previously shown to be enriched in RSKS-1/S6K mutants in *C. elegans*, suggesting it functions downstream of TOR signaling [50]. It is expressed in the intestine, similar to HZL-1, and levels of the protein also positively correlate with AMPK activity, as deletion of *argk-1* was shown to reduce levels of phosphorylated AAK-2. Thus, its prominence in our CLIP-seq data may indicate that regulation of *argk-1* mRNA by HZL-1 plays a role in the defects we see in *aak(0)* dauer larvae.

While the CLIP-seq analysis suggests that HZL-1 binds many mRNAs, it was unclear how these RNAs were being regulated. Some helicases can act as chaperones, protecting bound RNAs from degradation [51]. Conversely, other helicases can sequester mRNAs, inhibiting their function and/or degrading them directly [52]. We compared the HZL-1 RNA targets enriched specifically in the *aak(0)* dataset with our previous transcriptomic data. We found that many of the bound targets of HZL-1 are decreased in the *aak(0)* background dauer compared to *daf-2* controls (Fig 5C). This pattern is inverted when looking at the *aak(0); hzl-1* dataset, i.e., expression of those genes is increased relative to *aak(0).* This suggests that the levels of those RNAs which are bound by wild-type HZL-1 are destabilized in the *aak(0)* mutants. Conversely, those RNAs that are expressed at a higher level in the suppressed *aak(0); hzl-1* mutants compared to *aak(0)* mutants are no longer destabilized by RNA binding by HZL-1*,* therefore resulting in hyperplasia and post-dauer sterility.

Based on this interpretation, we suspected that the elimination of at least some of these RNAs in the *hzl-1* mutant might revert the suppression, causing the animals to become sterile once more, as the pro-quiescence signal is lost. Indeed, performing individual RNAi against several of the highly enriched CLIP targets, such as *argk-1* and *hmit-1.1,* led to a reduction in post-dauer fertility of these *hzl-1* mutant animals (Fig 5D). We therefore conclude that these two HZL-1 targets, and potentially others, may constitute an ensemble of pro-quiescent signals that affect the dauer germ line, the downstream effects of which are compromised when the transcripts are bound by HZL-1. Notably, we did not observe any effect on fertility by performing RNAi on these targets in a *daf-2* control dauer background. This may indicate that *argk-1* and other targets are not the sole drivers of quiescence, and that other targets regulated by AMPK preserve quiescence in the dauer germ line in the absence of ARGK-1. This finding is consistent with our previous experiments demonstrating that the aberrant activation of HZL-1 through a non-phosphorylable mutant, which would presumably impinge on ARGK-1 activity or abundance, is not sufficient to affect fertility (Figs 2D–2F and S2B).

We used the intestinally expressed HZL-1 for our CLIP-seq experiments due to the increased abundance of the protein in this strain compared to endogenous expression. Although our data revealed several mRNA targets of *hzl-1* that we subsequently validated, we were curious whether other RNAs were being masked by our strain choice (*hzl-1* expression driven by a strong intestinal promoter). We therefore repeated the CLIP-seq experiment using large quantities of our endogenously expressed HZL-1 strain. Analysis of the bound RNAs revealed that in this context, HZL-1 in *aak(0)* dauer animals binds many mRNAs associated with the intestine and metabolism (Fig 5E), similar to what we see when HZL-1 is intestinally expressed (Fig 5B). There was a significant overlap in bound RNAs between the intestinal and endogenous datasets in *aak(0)* mutants (Fig 5F). Notably, this sequencing experiment also confirmed that endogenous HZL-1 binds *argk-1* and *hmit-1.1,* as these two RNAs were among the most enriched in our datasets: 1st and 2nd most enriched, respectively, in the endogenous dataset compared to 1st and 7th for intestinally-expressed HZL-1.

Given the abundance of mRNAs bound to HZL-1, which are presumably inhibited by the protein, we wondered whether HZL-1 uses its helicase function, as would be predicted from the primary sequence. Many helicases have roles associated with mRNA decay, including SMG-2 in *C. elegans*, which is involved in nonsense-mediated decay [53,54]. The HZL-1 homolog HELZ also binds and degrades RNAs in *Drosophila* [21]. These helicases generally possess conserved DEAD-BOX and related domains, which confer the ability to bind and interact with RNA substrates [55]. HZL-1 itself is predicted to possess a DEXXQ domain based on its amino acid sequence (Fig 1A). We tested the requirement of this domain by generating a CRISPR deletion mutant of *hzl-1* with this region removed, and then examined any potential reproductive phenotype, or other, that might arise as a consequence. We noted that *aak(0)* animals with the *hzl-1* variant mutation that lacked the helicase domain have increased fertility/brood size, and reduced germline hyperplasia (S5C–S5E Fig), similar to the *hzl-1* loss-of-function mutant. We thus conclude that HZL-1 requires its helicase domain for its function in regulating the dauer germ line, ostensibly through its effects on select mRNAs.

### *argk-1* mediates dauer germline quiescence by influencing TOR activity

We observed that the knockdown of either *argk-1* or *hmit-1.1* resulted in the reversion of suppression of post-dauer sterility in *aak(0); hzl-1* mutants. Why these particular genes affect fertility, however, is unclear. *argk-1* appears to act downstream of S6K and thus TOR signaling [50]. *hmit-1.1* is an H+/myo-inositol transporter, implicated with the osmoprotective response in *C. elegans* [56]. Of the two, we were particularly intrigued by *argk-1* due its role in the regulation of metabolism, an aspect that is dramatically altered in AMPK mutants, and is often influenced by the intestine, the tissue where HZL-1 most likely functions.

**Fig 6:**
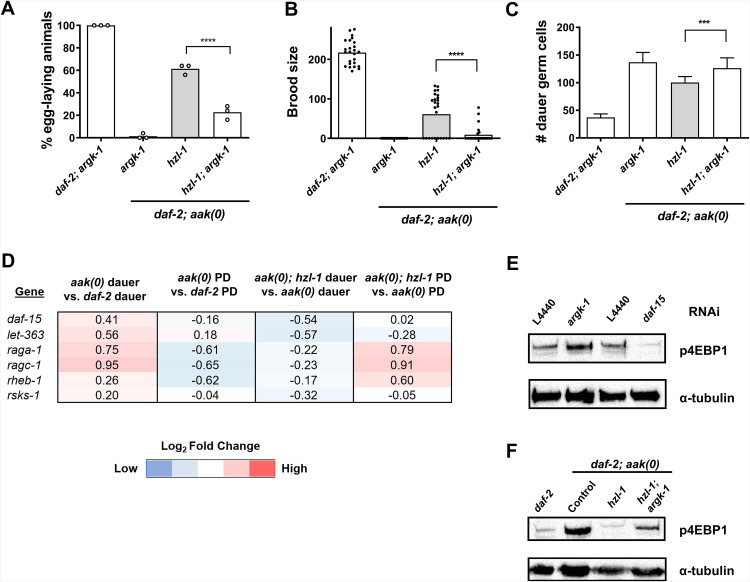
HZL-1 binds mRNAs such as *argk-1* to regulate post-dauer fertility. **A–C)** Post-dauer fertility, brood size, and germ cell counts of *argk-1* and *hzl-1* mutants. Post-dauer fertility data represent the results from three independent trials, where the mean is represented by columns and values for individual trials indicated by small circles. *n* = 50 for each trial. ****p < 0.0001 using one-way ANOVA for the indicated comparisons. All brood size assays and germ cell counts represent data from 25 individual animals per sample, with bars representing the mean and small circles representing individual values. ****p < 0.0001 using one-way ANOVA for the indicated comparisons. **D)** Heat map of fold change in TOR component genes obtained from RNA-seq datasets. Heat maps generated based on log2 fold change of indicated comparisons. **E)** and **F)** Levels of P-h4EBP1 used as a proxy for TOR activity detected by western blot in RNAi-treated animals or genetic mutants. Anti-P-h4EBP1 antibodies were used to detect phosphorylated h4EBP1 levels in animals. α-tubulin serves as a loading control **(E)** Dauer animals in *daf-;2 aak(0); hzl-1(0*) background treated with the indicated RNAi were used as samples. RNAi against *daf-15/*Raptor serves as a negative control. **F)** Dauer animals in indicated mutant backgrounds. Western analyses were performed on Day 2 dauer larvae for each experiment. Approximately ~600 dauer larvae were used for each well, run on an 8% SDS-PAGE gel for 45–90 min, as needed. P-h4EBP1 and α-tubulin bands are from the same respective gels, with membranes cut and separated after membrane transfer for simultaneous antibody incubation. The raw data underlying all figures can be found in S1 Data. Original blots can be found in S1 Raw Images.

We compared the phenotype of an *argk-1* loss-of-function mutation with the *argk-1* RNAi, and found that the deletion strain also affected post-dauer fertility and germline hyperplasia in the *aak(0); hzl-1* mutants (Fig 6A–6C), further confirming the influence of ARGK-1 on reproductive capability in these mutants. Loss of *argk-1* in *daf-2* control animals does not induce any reproductive defects, once again suggesting there may be compensatory mechanisms protecting dauer germline quiescence if AMPK is present. However, the loss of fertility in the *aak(0); hzl-1* mutant when *argk-1* is removed suggests that in AMPK mutants, *argk-1* mRNA is bound by HZL-1, and this targeting and subsequent inhibition contributes to post-dauer sterility.

We attempted to observe *argk-1* levels endogenously to understand how it might be functioning, but its expression proved difficult to observe, and we were only able to distinguish fluorescent signal around the pharynx (S6A Fig). Our observations of *argk-1* expression are consistent with previous attempts to image the protein [50], where it was shown to be in the pharynx and appeared to co-localize with glial cell markers. Notably, based on our observations, its expression in the dauer stage does not appear to be particularly distinct from its expression in other stages [50]. We further observed a subtle increase in expression levels between the different genotypes we tested, with more ARGK-1::mCherry signal detected in *daf-2* and *aak(0); hzl-1* mutants being more prominent compared to *aak(0),* consistent with our model where HZL-1 binds and modulates *argk-1* in the *aak(0)* background.

We further validated our model for the regulation of *argk-1* by carrying out RT-qPCR to determine the abundance of the mRNA in various strains. We observed high levels of *argk-1* in *daf-2* and *aak(0); hzl-1* mutant dauer larvae (relative fold changes of 1.0 and approximately 0.5, respectively), but lower levels in *aak(0)* animals (fold change of 0.2) (S6B Fig), consistent with our imaging analysis of the protein. We also saw higher levels of *argk-1*, ranging from around 0.5 to 0.75 relative fold change, in our various *hzl-1* mutants lacking IDRs or the helicase domain, or with the mutated S588 phosphoacceptor site. We thus confirm that HZL-1 function impacts the RNA levels of *argk-1,* such that when the helicase is impaired via mutation, it can no longer bind *argk-1* and thus the RNA levels are increased. This finding is consistent with our RNA-seq data (Fig 5C).

To validate that HZL-1 binds *argk-1,* we collected protein extracts from *daf-2* and *aak(0)* transgenic dauer animals possessing HZL-1::GFP, as we did for our CLIP-seq, and also collected extracts from the negative control strain expressing only GFP. We then performed immunoprecipitation using anti-GFP, and also with anti-FLAG as a non-specific antibody control, followed by RNA extraction (S6C Fig). *argk-1* levels were quantified by RT-qPCR analysis in these samples, and we observed high levels of the mRNA in the samples obtained from IP of HZL-1::GFP in the *aak(0)* background, relative to the lysate input (S6D Fig). Relative expression of *argk-1* was significantly higher in the GFP pulldown samples compared to the non-specific FLAG pulldown, confirming that *argk-1* mRNA was present specifically in HZL-1::GFP immunoprecipitates. This was true in our intestinally-expressed *hzl-1* strains, as well as in the immunoprecipitation we performed with endogenously-expressed *hzl-1.* Conversely, no significant *argk-1* levels were detected in the *daf-2* sample or the GFP control.

To further confirm that *argk-1* mRNA is bound by HZL-1, we also tested the S588 phosphomimetic variant of HZL-1, which we demonstrated has impaired function (Figs 2A–2C and S2A). Because we showed that modification of the phospho-site does not affect protein stability, we suspected it might inhibit the RNA-binding function of HZL-1. Consistent with this, our CLIP-RT-qPCR experiment showed that no significant levels of *argk-1* RNA could be detected within the GFP IP pellets isolated from the S588 phosphomimetic variant (S6D Fig). We therefore conclude that phosphorylation by AMPK at the residue S588 impairs the RNA-binding capacity of HZL-1, or at least its ability to bind *argk-1* mRNA, perhaps through inducing a conformational change, which has been demonstrated to affect this family of helicases [57].

While we demonstrated a clear link between HZL-1 and *argk-1* RNA, It is unclear how an arginine kinase may influence the germ line, but its functions downstream of TOR signaling may be relevant. The antagonistic interactions between AMPK signaling and the TOR pathway have been described extensively [58,59], and it is widely accepted that AMPK negatively regulates TOR to block its growth-enhancing effects during periods of energy stress [60]. Indeed, our own transcriptomic data confirms that the expression levels of many TOR components are increased in the AMPK mutant dauer larvae (Fig 6D). Curiously, ARGK-1 has also been shown to modulate AMPK, since active (S170 phosphorylated) AAK-2 was reduced in mutants that lack both ARGK-1 and S6K [50]. From these findings, it is probable that ARGK-1, AMPK, and TOR do not function in a simple linear relationship, but rather there exists the possibility of feedback loops or some other form of regulation (S6E Fig).

Our RNA-seq data indicate that the expression of *argk-1* negatively correlates with the expression of the major TORC1 components in our mutants (Figs 5C and 6D). It is therefore possible that ARGK-1 function indeed impinges on TOR activity. We monitored the effects of *argk-1* on a TOR activity sensor [61]. This *C. elegans* strain comprises a transgene expressing the human 4EBP1 protein, a well-characterized target that is phosphorylated by TOR. An antibody raised against the TOR-phosphorylated form of the protein can then be used to detect the modification as a proxy for TOR activity. The sensor transgene was then crossed into our various mutant backgrounds, and TOR activity was assayed simply through Western blotting using the anti-P-h4EBP1 antibody.

We performed *argk-1* RNAi in the *aak(0); hzl-1* mutant strain and picked transgenic animals to use as samples for western blot using the anti-P-h4EBP1 antibody. A stronger P-h4EBP1 signal was clearly visible in the sample from animals treated by the *argk-1* RNAi compared to those treated with the empty vector control (Fig 6E), suggesting that TOR activity is increased in response to the reduction of *argk-1* activity. To test the sensitivity of the sensor, we reduced TOR activity by eliminating the *C. elegans* orthologue of Raptor, *daf-15*, a key activating component in the TOR complex. As expected, we observed a weaker signal in animals treated with *daf-15* RNAi, indicating that the sensor faithfully reports TOR activity (Fig 6E). Furthermore, the sensor also indicated that TOR activity was high in *aak(0)* dauer larvae, but lower in both *daf-2* control dauer larvae and *aak(0); hzl-1* mutants (Fig 6F). These findings correlate with our RNA-seq data, which mirrors this elevated expression of TOR components in *aak(0)* animals (Fig 6D). Together, these data support a model whereby the levels of *argk-1* are able to alter TOR activity, and HZL-1 binding to *argk-1* RNA and inhibiting it in turn leads to elevated levels of TOR.

### TOR is active in the germ line of AMPK mutant dauer larvae and contributes to post-dauer sterility

Our model suggests that when *argk-1* RNA is bound and inhibited by HZL-1, as is the case in *aak(0)* animals, this leads to elevated TOR activity. We and others have found that TOR contributes to aberrant germline proliferation during periods of starvation during the L1 diapause in AMPK mutants [58,62], suggesting that a similar phenomenon may be occurring in the dauer stage as well.

To determine if misregulated TOR activation, specifically in the germ line of dauer *aak(0)* animals, influences the germline hyperplasia or has an impact on post-dauer fertility phenotypes in the AMPK mutants, we wanted to see how post-dauer fertility is affected when we inhibit TOR. We used an auxin-inducible degron (AID) system to circumvent the difficulties of using TOR mutants, which exhibit embryonic lethality or larval arrest phenotypes [18]. By fusing a degron sequence to DAF-15, a central component of TORC1 [63], we could effectively deplete TOR in various tissues, as has been previously described [64–66]. Furthermore, we were able to take advantage of tissue-specific TIR expression to eliminate the protein in a spatially and temporally controlled manner.

**Fig 7:**
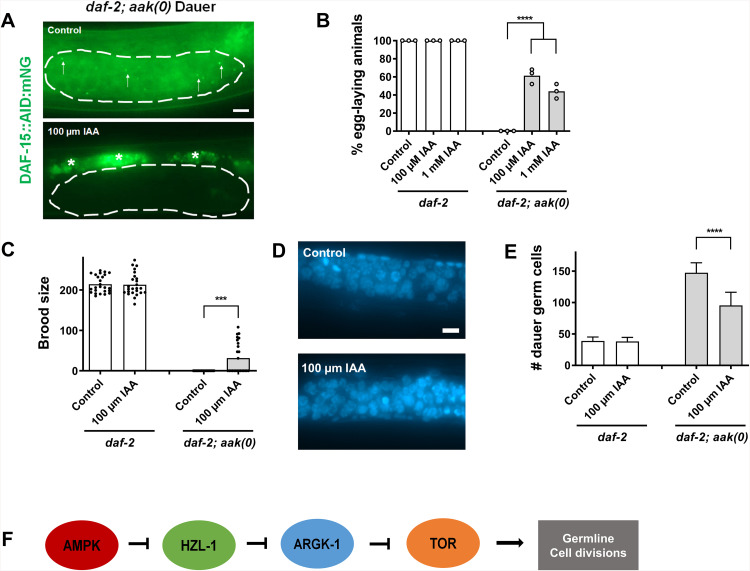
HZL-1 mRNA binding reverses *argk-1-*dependent modulation of TOR activity. **A)** Confocal micrograph images of *daf-2; aak(0)* animals with DAF-15::mNG::AID, i.e., the DAF-15 degron. Animals grown on control plates (without auxin) (Top) express mNG in the germ line (within the white lines). DAF-15 localizes to discrete puncta, presumably lysosomes, as has been previously shown (white arrows). mNG signal is absent in animals exposed to 100 µm IAA (Bottom). White asterisks denote non-specific signal from autofluorescence. Scale bar = 10 µm. **B)** Post-dauer fertility of *daf-2* and *daf-2; aak(0)* animals with DAF-15:mNG::TIR and germline-expressed TIR following auxin treatment with either 100 µM or 1 mM IAA. Controls were grown on standard NGM plates without auxin during the dauer stage. **C)** Post-dauer brood size of *daf-2* and *daf-2*, *aak(0)* DAF-15 degron animals with and without auxin. **D)** Confocal images showing representative germ lines of dauer *daf-2* and *daf-2*, *aak(0)* DAF-15 degron animals with and without auxin, following DAPI staining. **E)** Quantification of germ cell counts in *daf-2* and *daf-2*, *aak(0)* DAF-15 degron animals with and without auxin, based on DAPI staining. Post-dauer fertility data represent the results from three independent trials, where the mean is represented by columns and values for individual trials indicated by small circles. *n* = 50 for each trial. ****p < 0.0001 using one-way ANOVA for the indicated comparisons. All brood size assays and germ cell counts represent data from 25 individual animals per sample, with bars representing the mean and small circles representing individual values. ****p < 0.0001 using one-way ANOVA for the indicated comparisons. **F)** Model of AMPK-mediated regulation of HZL-1 and TOR. In the wild type, AMPK inhibits HZL-1, allowing for HZL-1-target mRNAs, such as *argk-1*, to promote germ cell quiescence. *argk-1* negatively regulates TOR, and when *argk-1* is bound by HZL-1, TOR activity is elevated. This drives the unscheduled germ cell divisions and subsequent hyperplasia observed in AMPK mutants during periods of energy challenge, resulting in post-dauer sterility. The raw data underlying all figures can be found in S1 Data.

The AID possessing transgenic animals were exposed to auxin specifically during the dauer stage, and fertility was assayed in the post-dauer animals (protocol in S7A Fig). Expression of DAF-15, tagged to mNeonGreen, was largely absent in *daf-2* control dauer larvae, as expected. However, it was visible specifically in the germ line in *aak(0)* mutant animals (Fig 7A), consistent with our transcriptomic data. Expression in the germ line disappeared in animals exposed to auxin at either 100 µM or 1 mM, suggesting the degron system worked as intended. When assaying post-dauer fertility, we noted that loss of DAF-15 in the *daf-2* control background had no effect on fertility, but in the *aak(0)* animals, the addition of auxin improved fertility considerably, compared to controls (Fig 7B and 7C), suggesting elevated TOR activity in the germ line was contributing to post-dauer sterility of AMPK mutants. We performed germ cell counts on these animals following DAPI staining and confirmed that germline hyperplasia was also significantly reduced in *aak(0)* dauer animals when DAF-15 is depleted (Fig 7D and 7E).

There were no changes in post-dauer fertility when DAF-15 was degraded in the soma (S7B Fig), suggesting that even if TOR is inappropriately active in somatic tissues, it has no negative impact on germline function in these animals. It should be noted, however, that it was difficult to perform this assay as soma-specific loss of DAF-15 induces developmental defects, including animals that are unable to recover from dauer, or dying prematurely (also noted by Schmeisser and colleagues, [66]). Furthermore, we note that while TOR disruption can result in developmental defects in *C. elegans* [18], degradation of DAF-15 in AMPK mutants specifically in the dauer germ line did not result in growth defects or arrest based on our observations.

We further tested our DAF-15 degron model in our *hzl-1* and *argk-1* mutants, and saw that the DAF-15 signal is absent in *aak(0); hzl-1* dauer larvae but visible in *aak(0); hzl-1; argk-1* mutants (S7C Fig), consistent with our measurements of TOR activity in these strains (Fig 6C). Similarly, there are no changes in post-dauer fertility or other reproductive phenotypes when TOR is removed in the *aak(0); hzl-1* mutants, but the sterility seen in *aak(0); hzl-1; argk-1* mutants is partially suppressed by DAF-15 degradation (S7D–S7F Fig), suggesting the absence of ARGK-1 in this background once again leads to an upregulation of TOR activity that contributes to germ line defects. Furthermore, we observed no changes in *argk-1* mRNA levels in *daf-2; aak(0)* animals with DAF-15 depleted in the germ line (S7G Fig), consistent with our model which suggests *argk-1* regulates TOR, so that suppressing TOR later in the pathway has no effect on *argk-1* upstream. Our data therefore indicate that unchecked TOR activity in the germ line in the dauer stage of *aak(0)* mutants is one contributor to the post-dauer sterility defects, and this is modulated by *argk-1*, a HZL-1 mRNA target.

Taken together, these data suggest that AMPK suppresses HZL-1 in the dauer stage most likely through its phosphorylation of S588, thereby ensuring quiescence. When AMPK is absent, HZL-1 is able to bind and inhibit RNAs such as *argk-1*, and low levels of *argk-1* result in elevated TOR activity, which contributes to the observed defects in the germ line, culminating in post-dauer sterility (Fig 7F).

## Discussion

The ability of organisms to respond to changes in environmental conditions is crucial for their survival and reproductive fitness. *C. elegans* can enter the dauer diapause when faced with environmental stressors, and to ensure the animals are fertile post-dauer, the germ cells must enter a quiescent state to preserve the integrity of the germ line throughout this stage. Previously, we have shown that the loss of the metabolic regulator AMPK results in germline hyperplasia and post-dauer sterility, among a host of somatic defects, as well as reduced dauer survival [17]. AMPK allows organisms to adjust to energy stress, permitting cells/tissues to maintain homeostasis in the absence of sufficient nutrition. The kinase regulates a number of different targets, mostly through direct phosphorylation, and in its absence the misregulation of these factors render the animals incapable of adjusting to the developmental and metabolic challenges of the dauer diapause.

Here, we have identified a mechanism that *C. elegans* employs to adjust to fluctuating metabolic requirements through its ability to regulate RNA metabolism. We investigated how a previously undescribed, putative RNA-binding helicase blocks the activity of key RNAs that otherwise promote a quiescent state in the *C. elegans* germ line, and how that protein is in turn regulated by the highly conserved metabolic regulator AMPK. Through analysis of this helicase, we have identified yet another means through which developmental plasticity can be fine-tuned, allowing for animals to adopt different metabolic and developmental states depending on the environments they are confronted with. This further underscores the important role of RNA regulation as a major process by which organisms can control their metabolism, development, and ultimately, their reproductive fitness.

Our analyses indicate that this RNA-binding helicase, which we named HZL-1*,* functions in the intestine in order to exert its control over the germ line. This type of cross-tissue communication has been previously described in *C. elegans* [67,68]. Given the global transcriptomic changes and extensive remodeling that must occur during the dauer stage, it is not surprising that the animal responds to a localized input, in this case, nutrient status detected in the intestine, and is able to generate a response in other tissues, most importantly the germ cells. HZL-1 appears to target intestinal RNAs, such as *argk-1*, inhibiting them and their cognate genes to modulate downstream proliferation pathways, specifically TOR signaling, leading to growth (Fig 5). This likely occurs when animals are sufficiently fed and are on a trajectory towards reproductive development, whereas the pathway is suppressed in the dauer stage, when animals must be quiescent. When activated aberrantly, as seen in AMPK-deficient dauer larvae, this results in extensive proliferation of the germ cells. This finding highlights the necessity for tight regulatory control by factors such as AMPK, as an otherwise beneficial pro-growth signal can rapidly become deleterious for the organism if misregulated. Our evidence suggests that the activation of AMPK leads to the phosphorylation of HZL-1 at a single residue in order to inhibit the protein when needed (Fig 2). This form of regulation by AMPK has been observed previously [38,49,69], although the precise mechanism by which phosphorylation results in the modulation of HZL-1 and its function remains to be elucidated.

HZL-1 binds specific mRNAs, thereby affecting their stability and likely preventing their translation (Fig 5C). While the precise mechanism by which it affects its targets remains unclear, there is evidence to suggest the protein behaves similar to other, well-characterized RNA helicases. HZL-1 possesses predicted intrinsically disordered regions, a common feature of RNA helicases [70], and we demonstrated that removing two of the IDRs causes a reduction of function (Fig 3). The protein also contains a predicted helicase domain, and this too is essential for its role in regulating quiescence in the dauer germ line, as our CRISPR mutant of *hzl-1* lacking its DEXXQ domain had impaired function (S5C–S5E Fig).

We compared the protein structure of HZL-1 to other helicases that have a known function in the germ line or in regulating reproductive phenotypes, such as ZGRF1, which mediates meiotic recombination in mammalian models [25–26]. However, there exist significant differences with HZL-1, including the lack of certain key functional domains such as the Zinc finger, indicating the helicase in *C. elegans* has distinct roles. Indeed, we found no evidence that HZL-1 affects meiotic recombination, as there was no prevalence of dead embryos or higher incidence of male progeny in *hzl-1* mutants.

Many germ-line helicases in *C. elegans* bind RNA and are frequently associated with regulating small RNAs, such as ERI-6/7 [30], but our CLIP-seq experiments revealed that HZL-1 does not bind small RNAs, but rather mRNAs. Given its role in mRNA degradation, HZL-1 may be closer in function to RNA helicases associated with nonsense-mediated decay, including SMG-2 [53], or indeed its homolog in other species HELZ [21]. Further study is required to elucidate the precise mechanism of HZL-1 and how it interacts with its target RNAs. One avenue may be to determine whether HZL-1 interacts or acts in concert with known factors associated with the NMD pathway.

The transcriptome of *aak(0); hzl-1* mutants differs dramatically from animals lacking AMPK alone, resembling the gene expression profile of wild-type animals. Many genes associated with reproduction and germline homeostasis are enriched in *aak(0)* dauer larvae, but are decreased in the *aak(0); hzl-1* mutant background (Fig 4B). This is consistent with previous findings which suggest AMPK-deficient dauer larvae skip the quiescence typical of the stage and adopt a reproductive state instead. This leads to premature or discordant germ cell development. Indeed, the dauer transcriptome of *aak(0)* mutants is strikingly similar to the post-dauer transcriptome of wild-type animals (Figs 4D and S4C). In the absence of AMPK, *hzl-1* and other factors could contribute to this by activating growth pathways that ultimately result in widespread gene misregulation. As a result, when HZL-1 itself is disabled, there is a partial return to wild-type gene expression.

A CLIP-seq experiment revealed HZL-1 binds RNAs, specifically mRNAs, similar to other RNA helicases. It inhibits its target RNAs, perhaps through degradation or sequestration [52]. This hypothesis is supported by our observation that many mRNA targets of HZL-1 have reduced expression in *aak(0)* dauer larvae, but have increased expression in the *aak(0); hzl-1* mutants (Fig 5C). It is unclear, however, how its RNA targets exert an effect on dauer physiology. It is plausible that some are responsible for the distinct survival defect of the mutant [49] as several are implicated in metabolic processes (Fig 5A and 5B). We have previously identified a number of genes that, when compromised, suppress the reduced survival defect of *aak(0)* dauer larvae [71], and many of these are metabolic genes. Notably, we detected RNAs bound to HZL-1 in both control and *aak(0)* backgrounds, although there were more RNAs bound in the AMPK mutants, where HZL-1 is presumably more active. It is unclear what function, if any, HZL-1 is carrying out in wild-type dauer animals.

*argk-1* stood out as the most enriched target bound to HZL-1 in *aak(0)* mutant dauer larvae. This arginine kinase is also the most enriched protein observed in S6K/*rsks-1* mutants in adult *C. elegans* based on mass spectrometry analysis [50], suggesting it is negatively regulated by *rsks-1* and thus acts downstream of, or in parallel to, TOR signaling [72,73]. Curiously, *argk-1* was also shown to be necessary for the phosphorylation and activation of AAK-2 in the *rsks-1* mutant background, a process that is required for the increased longevity of the *rsks-1* animals. We revealed that the loss of *argk-1* reverts the suppression of post-dauer sterility in *hzl-1* mutants (Fig 6A). It came as a surprise that the protein could have such a distinct role in reproductive development, given its function in other species, as creatine kinase has been more directly linked to energy modulation [74]. Our RNA-seq data suggested high TOR activity was a phenotype of AMPK dauer larvae (S6D Fig), and we confirmed a role of TOR regulation through the use of a TOR activity sensor, with which we saw an increase in TOR activity following a knockdown of *argk-1* (Fig 6E), suggesting ARGK-1 is a negative regulator of TOR in this developmental context.

It remains unclear how ARGK-1 is able to influence TOR activity, specifically in the germ line, but there is evidence that this regulation may be indirect. In mammals, creatine kinase, the closest ortholog of ARGK-1, is an enzyme that serves as an ATP generator [75]. The ability of creatine kinase to affect metabolism may be conserved in *C. elegans*. If that is the case, it is perhaps not surprising that TOR activity, which fluctuates in response to various metabolic changes, is also affected by the activity of ARGK-1. Indeed, studies have demonstrated that changing the levels of creatine kinase can have an impact on TOR activity. Specifically, overexpression of the creatine kinase brain (Ckb) isoform in cells downregulates mTOR [76], consistent with our findings of ARGK-1 being a negative TOR regulator. Conversely, another study demonstrated that loss of Ckb reduced CD8^+^ T cell expansion because of weakened mTOR signaling [77]. The complex relationship between creatine kinase activity and the TOR pathway is likely indicative of different metabolic responses that are required in specific tissues or other conditions.

There are notable physiological differences between these mammalian cell models and the *C. elegans* dauer model we have examined, and it is also not well established at the biochemical level how similar the ARGK-1 and mammalian creatine kinase might actually be. Nevertheless, there is established precedent that these enzymes can affect the action of the TOR pathway, and further metabolomic analyses could potentially clarify the specific function of ARGK-1 and its role in metabolic regulation.

The role of the TOR complex in regulating longevity, stress resistance, and other related phenotypes have been thoroughly studied, particularly in *C. elegans* [18,72,78–80]. However, it also plays a role in maintaining fertility, in nematode species and other models [65,81]. Our work provides yet another example of how TOR activity must be tightly controlled, specifically in a period of acute metabolic stress, in order to preserve reproductive fitness. Previously, it has been demonstrated that increased TOR activity contributes to aberrant germline proliferation in L1-starved *aak(0)* animals [58]. Our degron experiment, where we depleted TOR specifically in the germ line of dauer *aak(0)* mutants, also indicated that TOR may function in a similar manner in the dauer stage (Fig 7).

Regulation of TOR by AMPK has been well-studied [32,82], and so it is not surprising that TOR activity contributes to post-dauer phenotypes in AMPK-deficient mutants. TOR is one of several metabolic regulators which controls the growth of organisms, and thus must be modulated during the quiescent dauer phase. Without AMPK serving as a master regulator, however, TOR activity is elevated, leading to deleterious developmental defects as the animal can no longer reconcile its continued growth with the nutritional limitations of the dauer stage. This revelation highlights the importance of coordinating development with metabolic status. Animals enter the dauer stage when faced with harsh environmental conditions, such as a lack of nutrients, and a subsequent downregulation of developmental programs is critical such that the meager resources available are not used inappropriately, and the organism can instead survive until conditions improve.

Our work identifies a throughline between the metabolic regulator AMPK and the maintenance of germline quiescence in the dauer stage through the inhibition of TOR signaling (Fig 7F). Rather than direct regulation, however, we identify a potentially rapidly reversible mechanism by which HZL-1, a putative RNA helicase, modulates levels of various RNAs in the intestine in the absence of AMPK, resulting in widespread changes to the transcriptome, and activation of downstream signaling pathways, including TOR. Specifically, the inhibition of a metabolic gene, *argk-1*, contributes to the disruption of germline quiescence. Our unbiased genetic analysis of *hzl-1* has consequently expanded our understanding of the TOR signaling pathway. We demonstrated how the absence of regulators such as AMPK lead to strong TOR expression in the germ line even in the quiescent dauer stage, and how this directly impacts development and fertility. Most notably, we highlighted the role of a novel negative regulator of TOR in *C. elegans*, ARGK-1, although the precise mechanism through which this regulation might be executed remains to be fully elucidated.

## Materials and methods

### Maintenance of C. elegans strains

Animals were grown on nematode growth medium (NGM) plates seeded with *E. coli* OP50. *C. elegans* strains with the *daf-2(e1370)* mutation were maintained at 15 °C to ensure no dauer formation. Synchronization and sterilization of *C. elegans* embryos were performed using sodium hydroxide and sodium hypochlorite solutions, using standard protocols [83]. For most experimental procedures, animals were bleach-synchronized and allowed to hatch into L1 larvae before being dispensed onto plates, in order to ensure all animals were of the same developmental stage. Transgenic animals were maintained by picking rollers

List of all *C. elegans* strains used is listed in S1 Table.

### Proteomic analysis

Putative AMPK phosphorylation sites were identified using GPS 6.0 [84]. *C. elegans* proteome sequences were inputted into the program, and putative p-sites were identified using the species-specific batch predictor module, using the high threshold setting. Putative targets were further screened based on relevance to RNA pathways or functions. A relevant section of this proteomic analysis is detailed in a supplementary Excel file, S2 Raw Data.

Alphafold 3 was used to predict and analyze protein structures [37].

### RNA interference experiments

RNAi experiments were performed using feeding, as described [85]. Briefly, dsRNA-producing bacteria was grown with Ampicillin selection overnight at 37 °C using LB, then seeded onto NGM plates containing 1 mM IPTG and 50 µg/mL Ampicillin (i.e., “RNAi plates”) the following day. Plates were grown for a minimum of one day at room temperature before the addition of animals. For all RNAi experiments, L1 synchronized animals were put onto plates, then moved immediately to 25 °C to promote entry into dauer.

### Post-dauer fertility assay

Animals were kept at 25 °C for 4 days following L1 synchronization, then moved to 15 °C for further observation. Animals were picked onto either NGM plates, or plates growing RNAi identical to the plate being picked from, depending on the experiment in question. Singled animals were allowed develop for a minimum of 1 week, after which fertility was assayed by checking for the presence of F1 progeny. Three replicates were done for every post-dauer fertility assay.

### Brood size assay

Post-dauer animals were singled onto individual plates and allowed to lay eggs. Animals were moved to new plates as needed, when their progeny grew to L4. The total number of worms on each plate per singled hermaphrodite mother was counted. Twenty-five individual animals had their brood size assayed per condition.

### Confocal microscopy

2% agarose microscopy pads were created on microscope slides, and 20 mM levamisole (500 mM when mounting dauer animals) was deposited onto cooled agarose. Animals were picked onto slides and allowed to sit for approximately five minutes to induce paralysis, before being covered by a slide cover. Edges of slide covers were sealed using nail polish as needed. Slides were viewed on a Leica DMI 6000B inverted microscope equipped with a Quorum WaveFX spinning Disc and EM CCD, under various magnifications with and without oil immersion. Low-resolution images were obtained through a single stack, while high-resolution images were taken through a Z stack with a range of 10−5 µm and intervals of 0.2 µm. Images were processed via deconvolution and maximum projection for the Z stack, and further processed as needed using the application Fiji.

### DAPI staining and germ cell count

Dauer larvae were collected after 96 hours of growth at 25 °C. Animals were washed into tubes using M9 and aspirated, and 500 µl Carnoy’s solution (60% ethanol, 30% acetic acid, 10% chloroform) was added to the pellet before being rotated overnight at 4 °C.

The following day, samples were washed with phosphate-buffered saline with tween-20 (PBST) (1×PBS with 0.1% Tween 20) thrice, then 0.1 mg/ml DAPI solution was added to pellets. Samples incubated at room temperature for 30 min with gentle rotation. Following this, four washes were carried out with PBST, with a 15 min room temperature rotating incubation in PBST between each wash. The samples were then rotated at room temperature for 30 minutes. After the final wash, samples were aspirated until approximately 20 µl liquid remained, and then dispensed onto clean glass slides. Each sample was covered with a cover slip and the edges sealed with nail polish. Samples imaged using confocal microscopy, as described above and stored at 4 °C.

For germ cell counts, a population of animals for each condition was subjected to DAPI staining. Individual animals were observed under 63× zoom using a confocal microscope, with germ cells distinguishable with DAPI signal. The number of germ cells in 25 animals per condition was counted, going through the *Z* axis to ensure all germ cells were accounted for.

### Male frequency assay

Individual hermaphrodites were singled and allowed to lay eggs, being moved to new plates as needed when their progeny reached the L4 stage. The number of males produced by each individual animal was scored against the total brood size. 3 replicate experiments were carried out per strain, with 5 hermaphrodites per strain used in each replicate.

### Generation of transgenic lines

High-fidelity PCR was performed to amplify gene fragments of interest from *C. elegans* worm lysis, as well as to linearize relevant plasmid vectors. Constructs were generated through Gibson assembly [86] and following transformation into competent cells, cracking was carried out, and gel electrophoresis performed to identify colonies with plasmid DNA of the correct size.

Plasmids were microinjected into *C. elegans* strains of interest along with a *rol-6* marker for selection. Concentrations of ~30 µl was used for the selection marker and ~120–160 µl for the plasmid of interest, diluted in ultrapure water. F2 transformants were selected by picking rollers and maintained as independent lines.

### CRISPR editing

CRISPR editing was carried out as described previously [87]. Briefly, RNP complexes were assembled via 15 min incubation at 37 °C using the following ratio:

Cas9: 30 pmol
tracrRNA: 90 pmol
crRNA: 95 pmol

Subsequently, the injection mixture was prepared as follows:

RNP complex with Cas9 mix
Repair template: 100 ng/µl
*rol-6* injection marker plasmid: 40 ng/µl

For microinjection, healthy, well-fed young adult animals were picked onto agarose pads with oil, and both gonad arms were injected. Animals were then picked onto NGM plates, and a small volume of M9 added to each worm, with the oil being cleaned off.

Injected animals were allowed to lay eggs, and roller progeny were picked onto separate plates. After these animals laid eggs, they were genotyped to identify worms containing the mutation of interest. Progeny from plates that had the mutation were singled and allowed to propagate, and were then subjected to PCR genotyping to identify homozygotes.

All relevant oligos used in CRISPR strain generation are listed in S2 Table.

### Western blot

Western Blotting was performed using standard protocol with 6%–15% SDS-Page gels, as needed. 20 µl of collected worm samples in phosphate-buffered saline (PBS), typically ~600 dauer larvae per sample, were loaded into wells and run at 85 V for the stacking gel, then at 100–150 V for the resolving gel, as required. Following membrane transfer, membranes were cut based on protein size, i.e., a single membrane might be cut in half at the 100 kDa mark in order to obtain one membrane containing bands at 50 kDa and another with bands at 150 kDa. Membranes were then incubated with either anti-GFP, anti-α-tubulin, or anti-P-h4eBP1 antibodies [61]. After overnight primary antibody incubation, membranes were washed with PBST 3 times, 15 min each, followed by secondary antibody incubation for 2 hours. After another 3 PBST washes, membranes were soaked with total 1 ml Clarity Western ECL Substrate, then imaged using the MicroChemi (DNR Bio Imaging Systems) and GelCapture software (Version 7.0.18), using exposure times of 0.5–30 s, as required.

For Phos-tag Western Blot, the protocol remained identical save for the addition of the Phos-tag acrylamide (Fujifilm Irvine Scientific 30493521) and MnCl_2_ during gel preparation. 6% SDS-Page gels were prepared as before, with the addition of 50 µl of 5 mM Phos-tag acrylamide dissolved in 3% methanol, as well as 50 µl of 10 mM MnCl_2_. 100 µl of water was removed from the solution to compensate for the added volume.

Raw images of all Western Blots presented or analyzed in this study are provided in a supplementary PDF file, S1 Raw Images.

### RNA extraction and sequencing

For RNA extractions, animals were washed with M9 and pelleted into Eppendorf tubes. Standard Trizol extraction [88] was performed on relevant samples to extract any present RNA.

Briefly, 200 µl of Trizol was added to 20 µl of packed worms, and tubes were subjected to freeze-thawing with liquid nitrogen at room temperature. After four freeze-thaws, samples were kept at 4 °C for approximately 1 hour to settle particulates, then briefly centrifuged at high speeds. The solution from each tube was then transferred to a second set of tubes, without disturbing the pellet. Fifty µl of chloroform was added to the second set of tubes, followed by 30 s of agitation by vortex mixer, 3 min of rest, then 15 min of centrifugation at high speeds. The aqueous phase from each tube was then isolated and placed into another set of tubes, at which point the chloroform extraction was repeated.

Following the second extraction of the aqueous layer, 125 µl of isopropanol was added to samples and then tubes were inverted, followed by several minutes rest, and then 10 min of centrifugation at high speeds. The liquid from tubes was aspirated without disturbing the pellet, and then samples were washed with 500–1,000 µl of 70% ethanol diluted in ultrapure water. After another 5 min centrifugation, solutions were aspirated as much as possible, and then tubes were air dried over the course of one hour, or as needed. Samples were dissolved in 10–20 µl ultrapure water at room temperature, and their approximate concentration measured using a Nanodrop. All centrifugation was carried out at 4 °C.

Following purification, total RNA from each sample was run on 1% agarose gel to confirm presence of 18s and 28s ribosomal RNA bands, confirming integrity. RNA was stored for <3 days at −80 °C before sending off for sequencing. Sequencing was performed using NextSeq Midoutput with 2x75bp coverage. For CLIP-seq, small RNA sequencing was carried out separately in addition to standard mRNA sequencing, with separate library preparation for each method.

### Analysis of RNA-seq data

RNA-seq data was analyzed using DESeq2. Volcano plots of RNA-seq data were created using a custom script in R Studio. Gene ontology was carried out using WormBase’s Gene Set Enrichment Analysis. Bubble plots, heat maps, and Venn diagrams were generated using custom R scripts, Microsoft Powerpoint, or online resources (https://www.bioinformatics.com.cn) [89]. Data analysis, thresholding, pairwise comparisons, etc. were performed using Microsoft Excel.

### Cross-linking immunoprecipitation

Large quantities of dauer worms from relevant strains were collected through washing with M9, and then were cross-linked with formaldehyde before being frozen at −80 °C. Samples were thawed and lysed using a sonicator, followed by immunoprecipitation.

### Preparation of beads

Immunoprecipitation was carried out using 25 mg Protein A beads per sample. 1 ml PBS was added to each sample of beads and allowed to swell for 1 hour, followed by brief centrifugation at low speeds, after which liquid was aspirated. Dilution buffer (PBS + 1% bovine serum albumin) was added to beads in a 1:1 ratio and tubes were rotated at 4 °C for 10 min. After centrifugation and aspiration of liquid, 12 µl of relevant antibody serum (GFP or FLAG) was added to each sample, followed by 1 hour rotation at 4 °C. Afterwards, beads were aspirated followed by another dilution buffer incubation and wash as before, followed by a PBS wash.

Then, freshly made dimethyl pimelimidate (DMP), created from 13 mg/ml DMP stock in 1 ml of wash buffer (0.2M triethanolamine in PBS) was added to tubes at a 1:1 ratio, followed by 30 min room temperature incubation. Beads were then washed with wash buffer with 5 min of rotation at room temperature before aspiration. DMP treatment and incubation was then repeated twice, with washing as before. After the 3rd treatment, quenching buffer (50 mM ethanolamine in PBS) was added to samples in a 1:1 ratio followed by 5 min of rotation at room temperature and then centrifugation and aspiration. Samples were then washed with elution buffer (1M glycine) with 10 min rotation at room temperature, and this step was then repeated. Finally, samples were washed with PBS + 0.1% Tween 3 times. At this point, beads were incubated with lysed samples as needed, rotating at 4 °C overnight.

The following day, all samples were centrifuged and aspirated, followed by 3 PBS washes. At this point, beads were aliquoted and used directly for Trizol RNA extraction (as described above).

### AID experiments

Auxin-supplemented NGM plates were created through addition of indole-3-acetic acid (IAA) from a 1 M stock solution to molten agar solutions once agar was sufficiently cooled. IAA was added to create plates with a working concentration of either 100 µM or 1 mM.

For AID experiments, animals were synchronized and grown on standard NGM plates, then picked onto Auxin plates for 48 hours at 25 °C, as described (S7A Fig), before being transferred to NGM plates for growth at 15 °C. As a negative control, degron-tagged animals were also grown on standard NGM plates without auxin. Depletion of the degron-tagged protein of interest was confirmed using confocal microscopy. Confocal imaging of animals from auxin plates, as well as post-dauer fertility assays, were performed as described above.

### RT-qPCR

Trizol RNA extraction was performed on washed worm samples, as described above. Approximately 1 µg of extracted total RNA was converted to cDNA using the High-Capacity RNA-to-cDNA Kit (AppliedBiosystems, 4387406). The 2× SyberGreen qPCR Master-mix (ZmTech Scientifique, Q2100N) was used for qPCR using 1–2 ng of cDNA. A Bio-Rad CFX384 Real-Time 96-well PCR qPCR Detection System (Bio-Rad) coupled with the CFX Maestro Software (Bio-Rad) was used to analyze qPCR data. *tba-2* served as a reference gene for calculations. Three replicates were performed for each qPCR reaction, with 1 additional replicate using DNAase-free H_2_O instead of cDNA. Fold change calculations were performed using the delta Ct method.

For CLIP-qPCR, immunoprecipitation was performed on relevant samples using both anti-GFP and anti-FLAG (i.e., a non-specific antibody). For qPCR, both antibody samples as well as the original input lysate were subjected to Trizol RNA extraction. For calculations, the % Input method was used. Briefly, the dilution factor of the original lysate (before IP) was taken into account (specifically, a dilution factor of 50), and relative expression was calculated compared to the lysate. The delta Ct method was used, whereby the adjusted Ct of the input was calculated by subtracting the Log_2_ of the dilatation factor from the Ct of each input. The delta Ct was calculated by subtracting the GFP and FLAG Ct values for each sample from the corresponding adjusted input Ct. Finally, % input was calculated as 2^-(adjusted Ct) for both the GFP and FLAG Ct values for each sample.

## Supporting information

S1 Fig RNAi analysis for putative AMPK targets reveals that the reduction of *parp-2* and *hzl-1* function is sufficient to suppress post-dauer sterility in *daf-2; aak (0)* animals. **A)** Post-dauer fertility in *daf-2* and *daf-2; aak(0)* animals following RNAi against putative AMPK phosphorylation target genes, as predicted by GPS 6.0 software. **B)** Confocal images showing representative germ lines of *daf-2*, *aak(0)* and C44H9.4/*hzl-1* mutant dauer larvae following DAPI staining. **C)** Post-dauer fertility of control, *aak(0)* and *C44H9.4(0); aak(0)* animals treated with *parp-2* RNAi. L4440 serves as the empty vector control. Post-dauer fertility data represents three independent trials, with the mean represented by columns and values for individual trials indicated by small circles. *n* = 50 for each trial. ****p < 0.0001 using one-way ANOVA for the indicated comparisons. **D)** Percentage of males in indicated strains. Each circle represents three independent replicates, comprising approximately 500−1,000 animals spread over five plates per replicate following plating of an individual adult hermaphrodite. Animals were grown at the permissive temperature of 15 °C and the percentage of males in their brood were measured. All data was not significant based on ANOVA for indicated comparisons. The raw data underlying all figures can be found in S1 Data.
(TIF)

S2 Fig Phosphorylation at S588 attenuates HZL-1 function through mechanisms independent of differential degradation or localization. **A)** Post-dauer fertility of control animals, or those harboring transgenes that express phosphomimetic HZL-1 variants. Wild-type or mutated *hzl-1* was transgenically inserted into *daf-2; aak(0); hzl-1* strains. **B)** Post-dauer fertility of animals in control genetic backgrounds, or transgenics variants with non-phosphorylable mutations in the predicted AMPK consensus sites present in HZL-1. Wild-type or mutated *hzl-1* was introduced into *daf-2; hzl-1* strains as extrachromosomal arrays. All post-dauer fertility data represent three independent trials, with the mean represented by columns and values for individual trials indicated by small circles. *n* = 50 for each trial. ****p < 0.0001, ***p < 0.001 using one-way ANOVA for the indicated comparisons. **C)** Predicted protein models of HZL-1 wild-type or S588D phosphomimetic mutant, as generated by Alphafold 3 (Jumper and colleagues, 2021). Purple indicates the residue at position 588 and blue denotes the region around it. **D)** Closeup of HZL-1 wild-type and S588D mutant proteins from C), focused around the 588 residue and altered loop structure. Purple indicates the residue at position 588 and blue denotes the region around it. **E)** Levels of HZL-1::GFP detected by western blot using anti-GFP antibodies. Western analysis done with phosphomimetic HZL-1 mutants compared with wild type (WT). α-tubulin (Bottom) is the loading control. All strains possess the *hzl-1* mutation rescued by transgenic insertion of *hzl-1* under the intestinal *nhx-2* promoter. Western analyses were performed on Day 2 dauer larvae for each experiment. Approximately ~600 dauer larvae were used for each well, run on a 6% SDS-PAGE gel for 45–90 min, as needed. All GFP and α-tubulin bands are from the same gel. Membranes were cut and separated after membrane transfer for simultaneous antibody incubation of GFP and α-tubulin. **F)** Quantification of HZL-1::GFP levels from the western blot in (B). Intensity of GFP bands were compared to α-tubulin bands from the corresponding sample, normalized to values from WT control. Background intensity was taken into account for each band. All data was not significant based on ANOVA for indicated comparisons. **G)** Confocal images of *nhx-2p*::*hzl-1*::GFP in *daf-2; aak(0); hzl-1* mutants, with either WT *hzl-1* (Top) or S588D phosphomimetic *hzl-1* (bottom) in the transgene. Scale bar = 10 µm. The raw data underlying all figures can be found in S1 Data. Original blots can be found in S1 Raw Images.
(TIF)

S3 Fig Removal of IDR sequences does not affect *hzl-1* mRNA levels. **A)** RT-qPCR quantification of *hzl-1* mRNA levels in dauer *daf-2; aak(0); hzl-1* mutants that harbored variants of the reverting transgene *nhx-2p::hzl-1,* that had one or more IDR sequences deleted from the *hzl-1* sequence. Statistical significance was tested using *t* test. **B)** Post-dauer fertility of *daf-2; aak(0); hzl-1* rescued with *nhx-2p*::*hzl-1* with deletion variants of single or compound IDR deletions (ΔIDR1, ΔIDR2 and ΔIDR3) from the HZL-1 sequence. “Control” refers to intact wild-type HZL-1 expressed in the gut under the control of the *nhx-2* promoter. Post-dauer fertility data represents the results from three independent trials, where the mean is represented by columns and values for individual trials indicated by small circles. *n* = 50 for each trial.****p < 0.0001 using one-way ANOVA for the indicated comparisons. The raw data underlying all figures can be found in S1 Data.
(TIF)

S4 Fig Protocol for our RNA-seq. **A)** Protocol of RNA-seq methodology. Animals of the indicated genotypes were grown in large quantities at 25 °C until reaching day 2 of the dauer stage, and were either harvested for the dauer sample, or allowed to grow at 15 °C for another 4 days before harvesting for post-dauer samples. Total RNA was obtained through Trizol extraction before being sent to a sequencing facility. mRNA sequencing was performed, followed by reference genome assembly. DESeq2 was used for bioinformatic analysis. B) Volcano plots depicting the spread of gene expression changes between *aak(0); hzl-1 and aak(0)* dauer and post-dauer (PD) animals. **C)** and **D)** Bubble plots depicting most enriched GO terms in *aak(0)* animals compared to *daf-2* controls (C), and *hzl-1(0); aak(0)* compared to *aak(0)* (D), ranked by significance based on p-value. C) Left and right graphs represent dauer and post-dauer comparisons, respectively. Top graphs depict genes increased in *daf-2; aak(0)* mutants compared to *daf-2* controls, while bottom graphs depict those genes that were decreased. Size of bubbles indicate number of genes in their respective categories that were enriched in the dataset. GO enrichment was conducted using the Wormbase Gene Set Enrichment Analysis [46,47]. The raw data underlying all figures can be found in S1 Data.
(TIF)

S5 Fig Protocol of CLIP-seq methodology and tissue/phenotype enrichment of CLIP-seq data. **A)** Protocol of CLIP-seq methodology. Animals of the indicated genotypes were grown in large quantities at 25 °C until they reached day 2 of the dauer stage, and then were subjected to formaldehyde cross-linking, followed by immunoprecipitation with an anti-GFP antibody. Trizol RNA extraction was subsequently performed on the immunoprecipitated proteins. mRNA and small RNA sequencing was performed on the total RNA obtained, followed by reference genome assembly. **B)** Bubble plots depicting Top 100 RNAs bound to HZL-1 in the *daf-2* control background, ranked by significance based on p-value. Size of bubbles indicate number of genes in their respective categories that were enriched in the indicated dataset. GO enrichment analysis was conducted using the Wormbase Gene Set Enrichment Analysis [46,47]. **C–E)** Post-dauer fertility, brood size and germ cell count of *daf-2; aak(0)* mutants with the *hzl-1* helicase domain deletion. Post-dauer fertility data represent the results from three independent trials, where the mean is represented by columns and values for individual trials indicated by small circles. *n* = 50 for each trial. ****p < 0.0001 using one-way ANOVA for the indicated comparisons. All brood size assays and germ cell counts represent data from 25 individual animals per sample, with bars representing the mean and small circles representing individual values. ****p < 0.0001 using one-way ANOVA for the indicated comparisons. The raw data underlying all figures can be found in S1 Data.
(TIF)

S6 Fig Levels of *argk-1* mRNA are regulated by HZL-1. **A)** Confocal images depicting ARGK-1::mCherry expression in the head region of indicated strains. Images taken at 63× zoom under oil immersion. Scale bar = 10 µm. **B)** RT-qPCR data of *argk-1* mRNA levels in dauer animals of indicated strains. ***p < 0.005, **p < 0.05 as determined by multiple *t* test. All statistical analyses carried out against the “Control’ dataset. **C)** Protocol for CLIP-RT-qPCR. Animals of the indicated genotypes were grown in large quantities at 25 °C until they reached day 2 of the dauer stage, and then were subjected to formaldehyde cross-linking, followed by immunoprecipitation with an anti-GFP antibody. Anti-FLAG was used as a non-specific control. Trizol RNA extraction was subsequently performed on the immunoprecipitated proteins followed by cDNA conversion and RT-qPCR using primers for *argk-1.* S588D refers to the phosphomimetic variant of HZL-1, which was expressed in place of the wild-type variant for that strain. GFP expressed under the intestinal promoter, but without *hzl-1*, served as a negative control. **D)** Quantification of CLIP-RT-qPCR experiment using RNA from either GFP or FLAG IPs. Primers against *argk-1* were used for RT-qPCR. All quantifications were carried out relative to Input, i.e., the lysate for the IP, taking into account a dilution factor of 2% 50 to determine the “Adjusted Input” value. % Input was calculated using the Delta Ct method. ***p < 0.0005 as determined by multiple *t* test. **E)** Model of the TOR pathway highlighting a potential modulatory role of ARGK-1. The raw data underlying all figures can be found in S1 Data.
(TIF)

S7 Fig TOR activity in the germ line is regulated by HZL-1 and ARGK-1 and contributes to post-dauer sterility of AMPK mutants. **A)** Protocol for using an auxin-inducible DAF-15 degron to assess the role of TOR, downstream of AMPK/HZL-1/ARGK-1. *daf-2; aak(0)* animals with DAF-15::mNeonGreen::AID and TIR expressed under a soma- or germline-specific promoter were grown on NGM plates for 48 hours at 25 °C, before being transferred to control or auxin-supplemented plates, also at 25 °C, degrade the protein specifically during the dauer stage. After 48 hours in dauer, some animals were picked for confocal imaging. The rest of the population was transferred to NGM plates and post-dauer fertility was assessed after they had recovered. **B)** Post-dauer fertility of *daf-2* and *daf-2; aak(0*) animals with DAF-15:mNG::TIR and soma-expressed TIR following auxin treatment with either 100 µM or 1 mM IAA. C) Confocal micrograph images of *aak(0); hzl-1* or *aak(0); hzl-1*; *argk-1* animals with DAF-15::mNG::AID. Animals grown on control plates (without auxin). Approximate region of germ line is shown between the white lines. DAF-15 enriched in puncta is also visible (white arrows). Asterisks denote non-specific signal from autofluorescence. Scale bar = 10 µm. **D–F)** Post-dauer fertility, brood size and germ cell count of *aak(0); hzl-1* or *aak(0); hzl-1*; *argk-1* animals with DAF-15:mNG::TIR and germline-expressed TIR following auxin treatment with 100 µM IAA. Controls were grown on standard NGM plates without auxin during the dauer stage. Post-dauer fertility data represents three independent trials, with the mean represented by columns and values for individual trials indicated by small circles. *n* = 50 for each trial. ***p < 0.001 using one-way ANOVA for the indicated comparisons. All brood size assays and germ cell counts represent data from 25 individual animals per sample, with bars representing the mean and small circles representing individual values. **p < 0.01 using one-way ANOVA for the indicated comparisons. **G)** RT-qPCR data of *argk-1* mRNA levels in dauer animals of indicated degron strains, with RNA harvested from control animals or animal treated with 100 µM IAA. All data not significant using *t* test. The raw data underlying all figures can be found in S1 Data.
(TIF)

S1 Data All relevant data values for figures. Excel file containing raw values for post-dauer fertility, brood size, germ cell count, qPCR, bubble plots, etc.
(XLSX)

S2 Data Putative AMPK targets as identified by GPS 6.0. Excel file containing summary of proteomic analysis, using GPS 6.0 to identify consensus AMPK target sites on select proteins. List of proteins derived from Wormbase “conserved_miRNA_and_siRNA_cluster”.
(XLSX)

S1 Raw Images Unedited original Western blots used in study. (PDF)

S1 Table List of *Caenorhabditis elegans* strains used in study. (PDF)

S2 Table List of oligonucleotides used in generation and validation of CRISPR mutants in study. (XLSX)

## Abbreviations

AID: auxin-inducible degron
AMPK: AMP-activated protein kinase
Ckb: creatine kinase brain
DMP: dimethyl pimelimidate
HZL-1: HELZ-like 1
IAA: indole-3-acetic acid
IDRs: intrinsically disordered regions
NGM: nematode growth medium
PBS: phosphate-buffered saline
PBST: phosphate-buffered saline with tween-20
TOR: target of rapamycin
