# Supplementary figures and images for: AMPK modulates a DEAH box RNA-helicase to attenuate TOR signaling and establish developmental quiescence in Caenorhabditis elegans
Source: PLoS Biol. 2025 Dec 1;23(12):e3003144. doi: 10.1371/journal.pbio.3003144 (PMC12685192; doi:10.1371/journal.pbio.3003144)

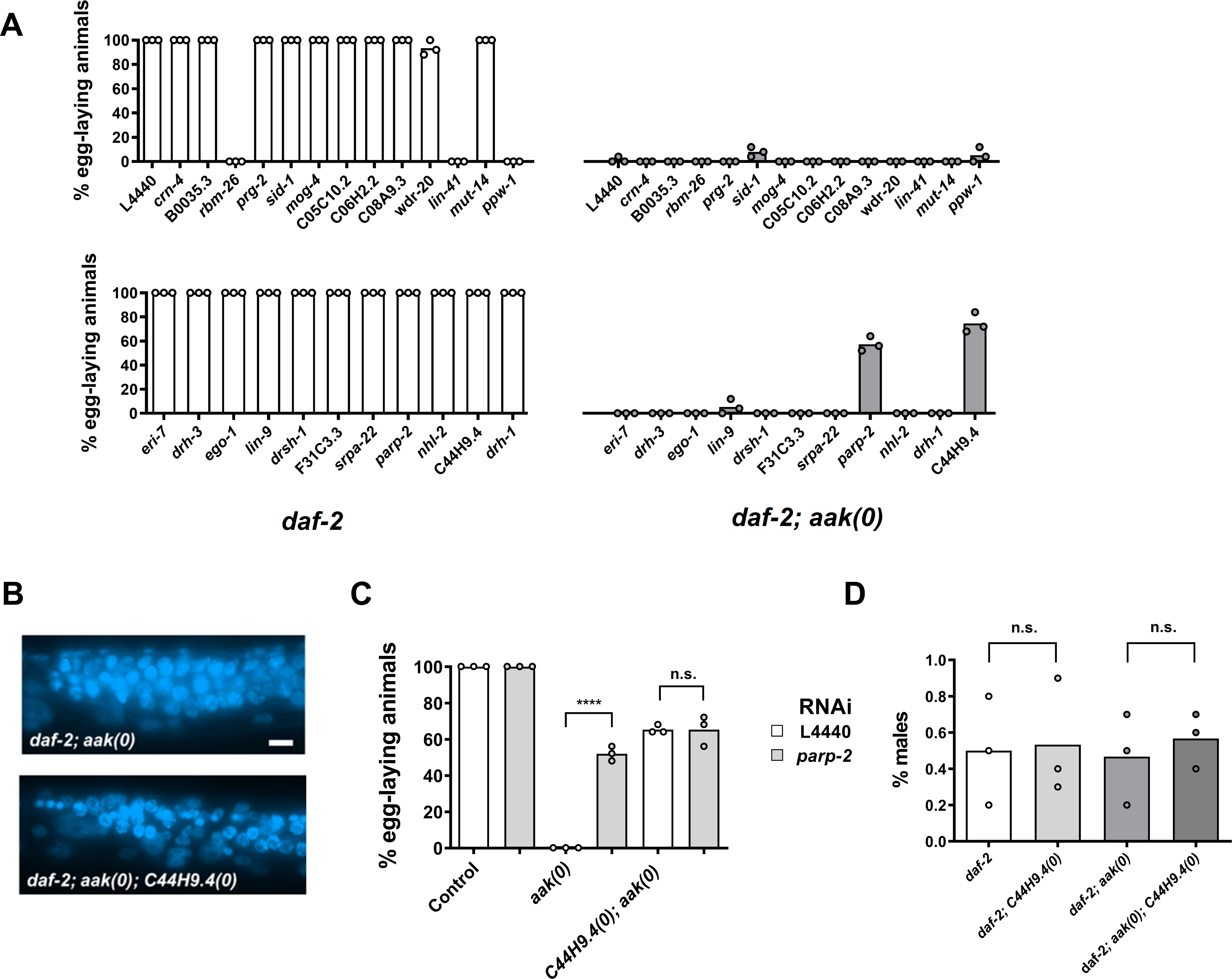

Supplement: S1 Fig — A) Post-dauer fertility in daf-2 and daf-2; aak(0) animals following RNAi against putative AMPK phosphorylation target genes, as predicted by GPS 6.0 software. B) Confocal images showing representative germ lines of daf-2, aak(0) and C44H9.4/hzl-1 mutant dauer larvae following DAPI staining. C) Post-dauer fertility of control, aak(0) and C44H9.4(0); aak(0) animals treated with parp-2 RNAi. L4440 serves as the empty vector control. Post-dauer fertility data represents three independent trials, with the mean represented by columns and values for individual trials indicated by small circles. n = 50 for each trial. ****p < 0.0001 using one-way ANOVA for the indicated comparisons. D) Percentage of males in indicated strains. Each circle represents three independent replicates, comprising approximately 500−1,000 animals spread over five plates per replicate following plating of an individual adult hermaphrodite. Animals were grown at the permissive temperature of 15 °C and the percentage of males in their brood were measured. All data was not significant based on ANOVA for indicated comparisons. The raw data underlying all figures can be found in S1 Data. (TIF) [file pbio.3003144.s001.tif]

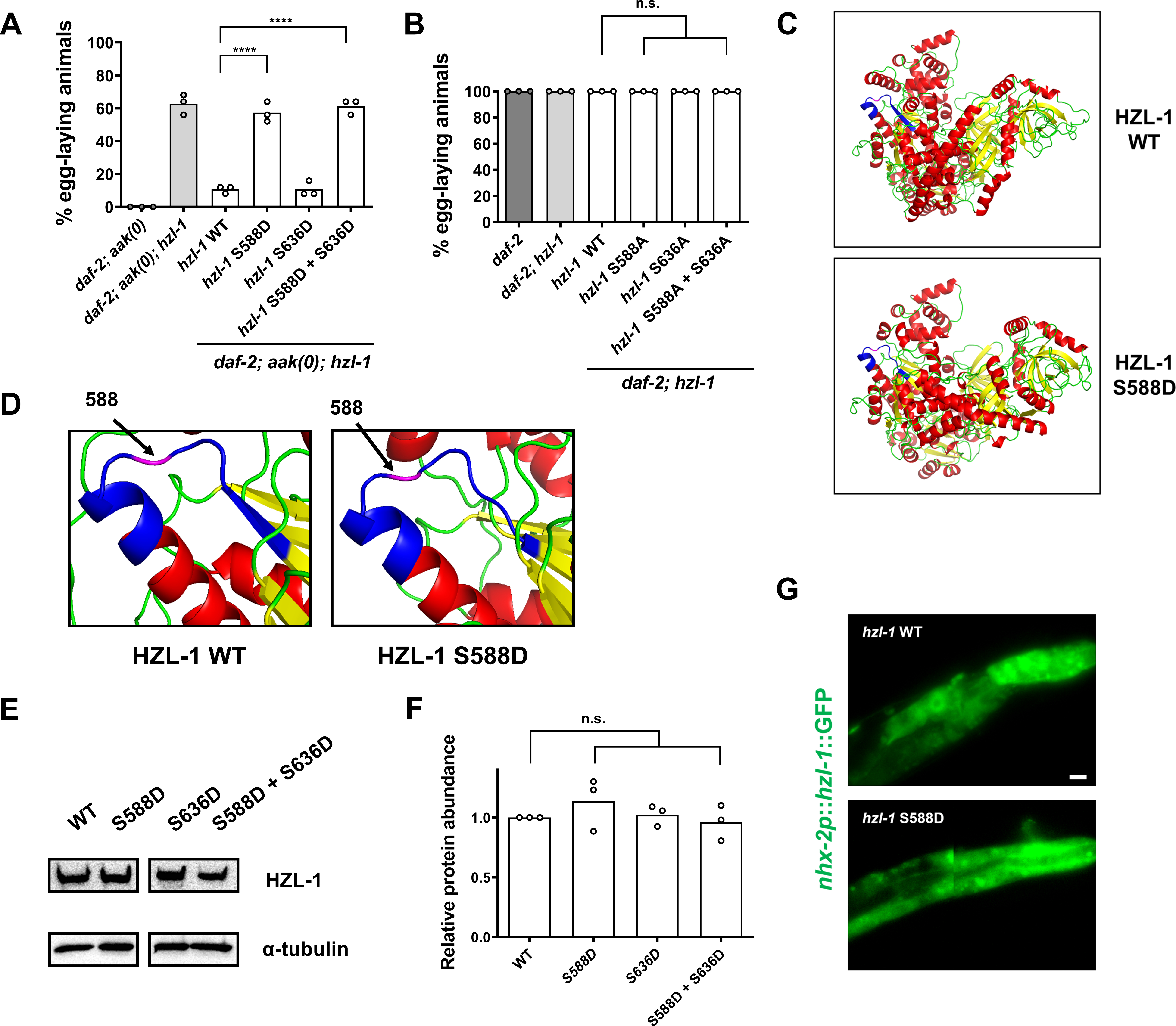

Supplement: S2 Fig — A) Post-dauer fertility of control animals, or those harboring transgenes that express phosphomimetic HZL-1 variants. Wild-type or mutated hzl-1 was transgenically inserted into daf-2; aak(0); hzl-1 strains. B) Post-dauer fertility of animals in control genetic backgrounds, or transgenics variants with non-phosphorylable mutations in the predicted AMPK consensus sites present in HZL-1. Wild-type or mutated hzl-1 was introduced into daf-2; hzl-1 strains as extrachromosomal arrays. All post-dauer fertility data represent three independent trials, with the mean represented by columns and values for individual trials indicated by small circles. n = 50 for each trial. ****p < 0.0001, ***p < 0.001 using one-way ANOVA for the indicated comparisons. C) Predicted protein models of HZL-1 wild-type or S588D phosphomimetic mutant, as generated by Alphafold 3 (Jumper and colleagues, 2021). Purple indicates the residue at position 588 and blue denotes the region around it. D) Closeup of HZL-1 wild-type and S588D mutant proteins from C), focused around the 588 residue and altered loop structure. Purple indicates the residue at position 588 and blue denotes the region around it. E) Levels of HZL-1::GFP detected by western blot using anti-GFP antibodies. Western analysis done with phosphomimetic HZL-1 mutants compared with wild type (WT). α-tubulin (Bottom) is the loading control. All strains possess the hzl-1 mutation rescued by transgenic insertion of hzl-1 under the intestinal nhx-2 promoter. Western analyses were performed on Day 2 dauer larvae for each experiment. Approximately ~600 dauer larvae were used for each well, run on a 6% SDS-PAGE gel for 45–90 min, as needed. All GFP and α-tubulin bands are from the same gel. Membranes were cut and separated after membrane transfer for simultaneous antibody incubation of GFP and α-tubulin. F) Quantification of HZL-1::GFP levels from the western blot in (B). Intensity of GFP bands were compared to α-tubulin bands from the correspondin [file pbio.3003144.s002.tif]

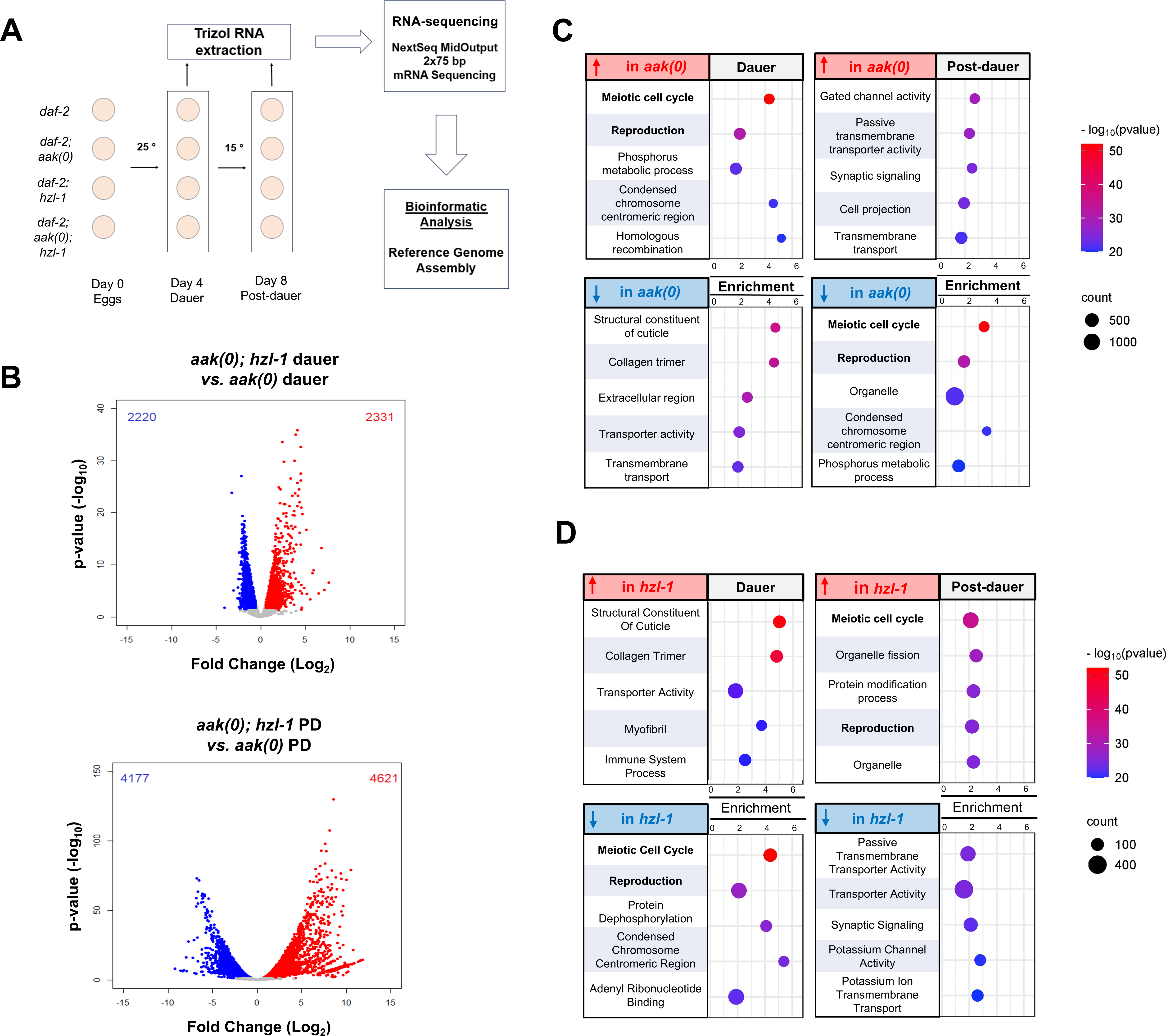

Supplement: S4 Fig — A) Protocol of RNA-seq methodology. Animals of the indicated genotypes were grown in large quantities at 25 °C until reaching day 2 of the dauer stage, and were either harvested for the dauer sample, or allowed to grow at 15 °C for another 4 days before harvesting for post-dauer samples. Total RNA was obtained through Trizol extraction before being sent to a sequencing facility. mRNA sequencing was performed, followed by reference genome assembly. DESeq2 was used for bioinformatic analysis. B) Volcano plots depicting the spread of gene expression changes between aak(0); hzl-1 and aak(0) dauer and post-dauer (PD) animals. C) and D) Bubble plots depicting most enriched GO terms in aak(0) animals compared to daf-2 controls (C), and hzl-1(0); aak(0) compared to aak(0) (D), ranked by significance based on p-value. C) Left and right graphs represent dauer and post-dauer comparisons, respectively. Top graphs depict genes increased in daf-2; aak(0) mutants compared to daf-2 controls, while bottom graphs depict those genes that were decreased. Size of bubbles indicate number of genes in their respective categories that were enriched in the dataset. GO enrichment was conducted using the Wormbase Gene Set Enrichment Analysis [46,47]. The raw data underlying all figures can be found in S1 Data. (TIF) [file pbio.3003144.s004.tif]

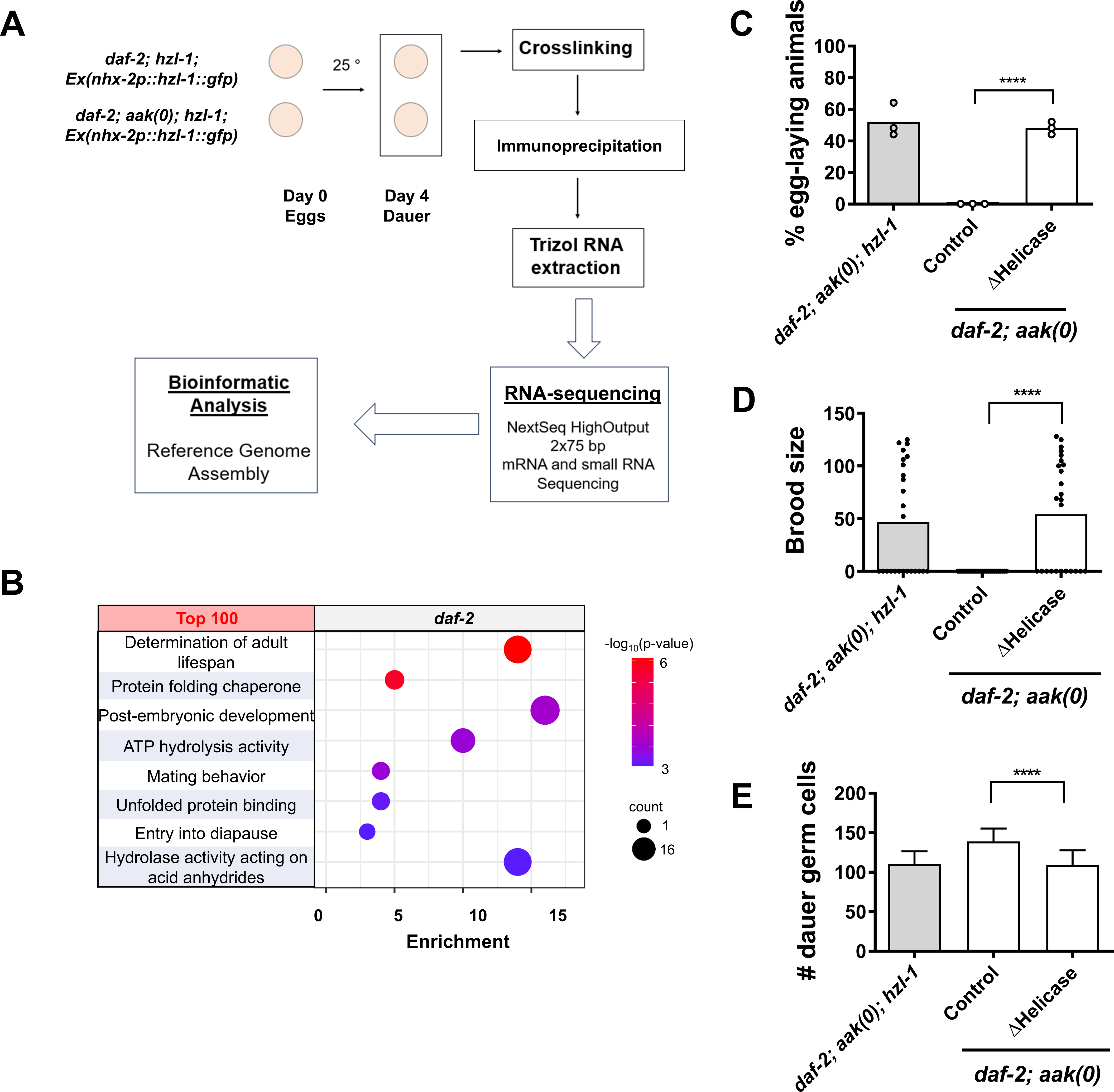

Supplement: S5 Fig — A) Protocol of CLIP-seq methodology. Animals of the indicated genotypes were grown in large quantities at 25 °C until they reached day 2 of the dauer stage, and then were subjected to formaldehyde cross-linking, followed by immunoprecipitation with an anti-GFP antibody. Trizol RNA extraction was subsequently performed on the immunoprecipitated proteins. mRNA and small RNA sequencing was performed on the total RNA obtained, followed by reference genome assembly. B) Bubble plots depicting Top 100 RNAs bound to HZL-1 in the daf-2 control background, ranked by significance based on p-value. Size of bubbles indicate number of genes in their respective categories that were enriched in the indicated dataset. GO enrichment analysis was conducted using the Wormbase Gene Set Enrichment Analysis [46,47]. C–E) Post-dauer fertility, brood size and germ cell count of daf-2; aak(0) mutants with the hzl-1 helicase domain deletion. Post-dauer fertility data represent the results from three independent trials, where the mean is represented by columns and values for individual trials indicated by small circles. n = 50 for each trial. ****p < 0.0001 using one-way ANOVA for the indicated comparisons. All brood size assays and germ cell counts represent data from 25 individual animals per sample, with bars representing the mean and small circles representing individual values. ****p < 0.0001 using one-way ANOVA for the indicated comparisons. The raw data underlying all figures can be found in S1 Data. (TIF) [file pbio.3003144.s005.tif]

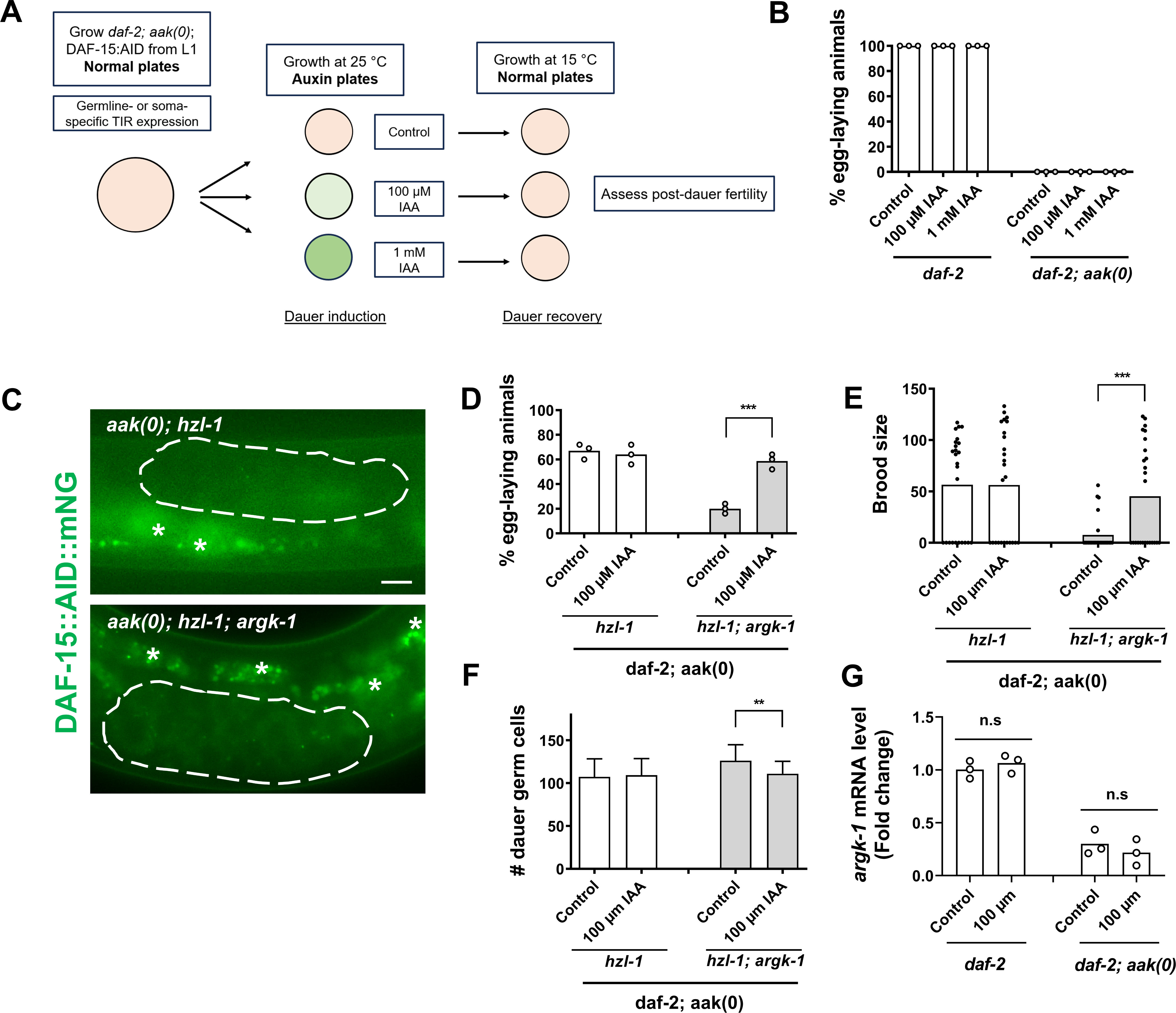

Supplement: S7 Fig — A) Protocol for using an auxin-inducible DAF-15 degron to assess the role of TOR, downstream of AMPK/HZL-1/ARGK-1. daf-2; aak(0) animals with DAF-15::mNeonGreen::AID and TIR expressed under a soma- or germline-specific promoter were grown on NGM plates for 48 hours at 25 °C, before being transferred to control or auxin-supplemented plates, also at 25 °C, degrade the protein specifically during the dauer stage. After 48 hours in dauer, some animals were picked for confocal imaging. The rest of the population was transferred to NGM plates and post-dauer fertility was assessed after they had recovered. B) Post-dauer fertility of daf-2 and daf-2; aak(0) animals with DAF-15:mNG::TIR and soma-expressed TIR following auxin treatment with either 100 µM or 1 mM IAA. C) Confocal micrograph images of aak(0); hzl-1 or aak(0); hzl-1; argk-1 animals with DAF-15::mNG::AID. Animals grown on control plates (without auxin). Approximate region of germ line is shown between the white lines. DAF-15 enriched in puncta is also visible (white arrows). Asterisks denote non-specific signal from autofluorescence. Scale bar = 10 µm. D–F) Post-dauer fertility, brood size and germ cell count of aak(0); hzl-1 or aak(0); hzl-1; argk-1 animals with DAF-15:mNG::TIR and germline-expressed TIR following auxin treatment with 100 µM IAA. Controls were grown on standard NGM plates without auxin during the dauer stage. Post-dauer fertility data represents three independent trials, with the mean represented by columns and values for individual trials indicated by small circles. n = 50 for each trial. ***p < 0.001 using one-way ANOVA for the indicated comparisons. All brood size assays and germ cell counts represent data from 25 individual animals per sample, with bars representing the mean and small circles representing individual values. **p < 0.01 using one-way ANOVA for the indicated comparisons. G) RT-qPCR data of argk-1 mRNA levels in dauer animals of indicated degron strains, with RNA harvested from [file pbio.3003144.s007.tif]
