# Supplementary material for: AMPK modulates a DEAH box RNA-helicase to attenuate TOR signaling and establish developmental quiescence in Caenorhabditis elegans
Source: PLoS Biol. 2025 Dec 1;23(12):e3003144. doi: 10.1371/journal.pbio.3003144 (PMC12685192; doi:10.1371/journal.pbio.3003144)
Supplement: S1 Raw Images — (PDF) [file pbio.3003144.s010.pdf]

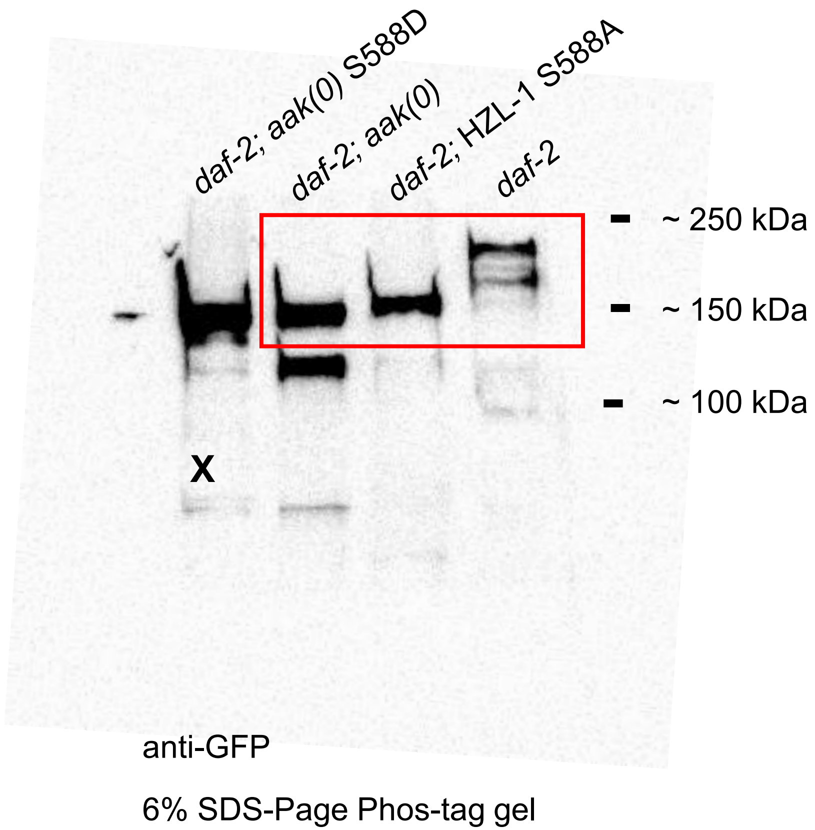

X – Lane not used in analysis
Red box – Lanes presented in manuscript figure

From Fig 2H) Representative Phos-tag Western blot depicting relative migration of HZL-1::GFP bands in indicated mutant strains, as detected by anti-GFP antibodies. *daf-2; aak(0)* mutant samples are compared with *daf-2* controls as well as *daf-2; hzl-1* mutants rescued with the S588A *hzl-1* variant. All strains possess the *hzl-1* mutation rescued by transgenic insertion of *hzl-1* under the intestinal *nhx-2* promoter.

Western analyses were performed on Day 2 dauer larvae for each experiment. Approximately ~600 dauer larvae were used for each well, run on a 6% SDS-PAGE Phos-tag gel for 45 to 90 minutes, as needed.

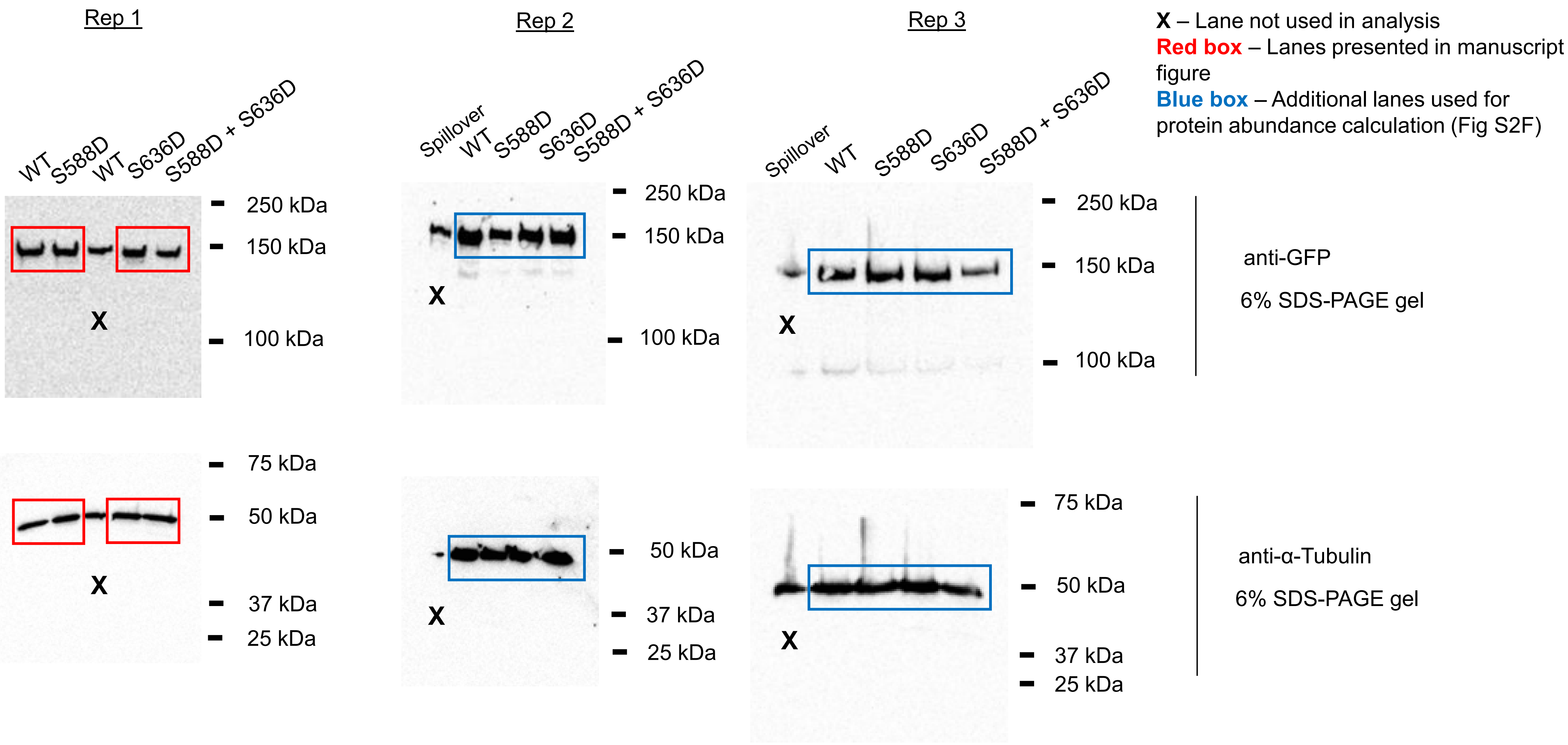

From Fig S2E) Levels of HZL-1::GFP detected by Western blot using anti-GFP antibodies. Western analysis done with phosphomimetic HZL-1 mutants compared with wild type HZL-1 (WT) in *daf-2; aak(0)* background. α-tubulin (Bottom) is the loading control. All strains possess the *hzl-1* mutation rescued by transgenic insertion of *hzl-1* under the intestinal *nhx-2* promoter.

Western analyses were performed on Day 2 dauer larvae for each experiment. Approximately ~600 dauer larvae were used for each well, run on a 6% SDS-PAGE gel for 45 to 90 minutes, as needed. All GFP and α-tubulin bands are from the same gel. Membranes were cut and separated after membrane transfer for simultaneous antibody incubation of GFP and α-tubulin.

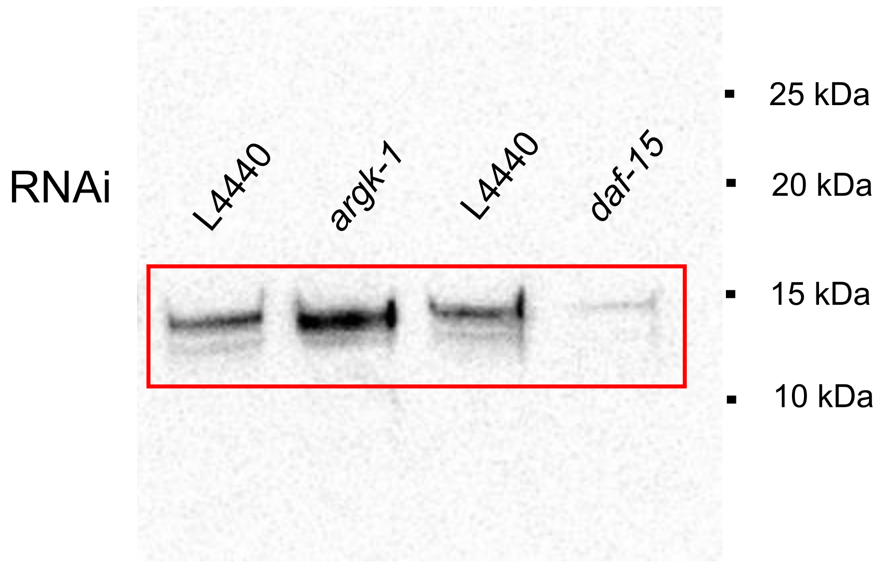

anti-h4EBP1

8% SDS-PAGE gel
Bottom half

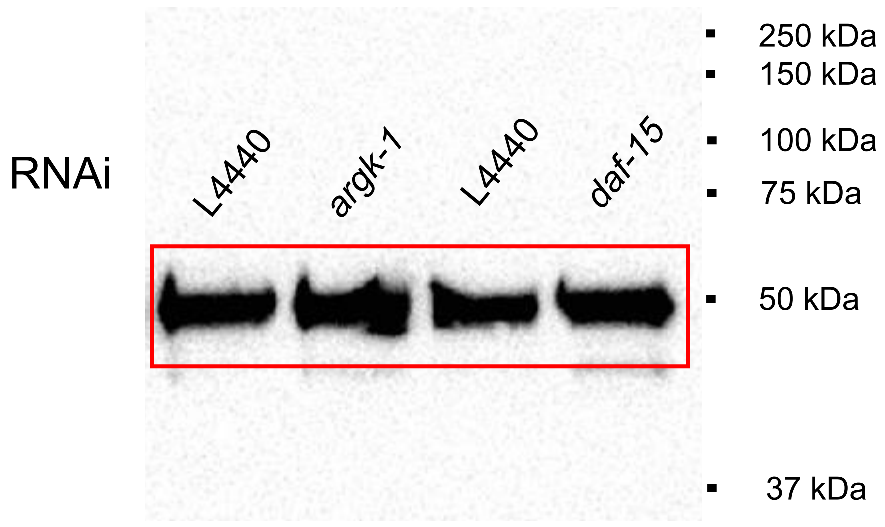

anti-α-Tubulin

8% SDS-PAGE gel
Top half

From Fig 6E) Levels of P-h4EBP1 used as a proxy for TOR activity detected by Western blot in RNAi-treated animals. Anti-P-h4EBP1 antibodies were used to detect phosphorylated h4EBP1 levels in animals. α-tubulin serves as a loading control. Dauer animals in *daf-;2 aak(0); hzl-1(0*) background treated with the indicated RNAi were used as samples. RNAi against *daf-15/*Raptor serves as a negative control.

Western analyses were performed on Day 2 dauer larvae for each experiment. Approximately ~600 dauer larvae were used for each well, run on an 8% SDS-PAGE gel for 45 to 90 minutes, as needed. P-h4EBP1 and α-tubulin bands are from the same respective gels, with membranes cut and separated after membrane transfer for simultaneous antibody incubation.

X – Lane not used in analysis
Red box – Lanes presented in manuscript figure

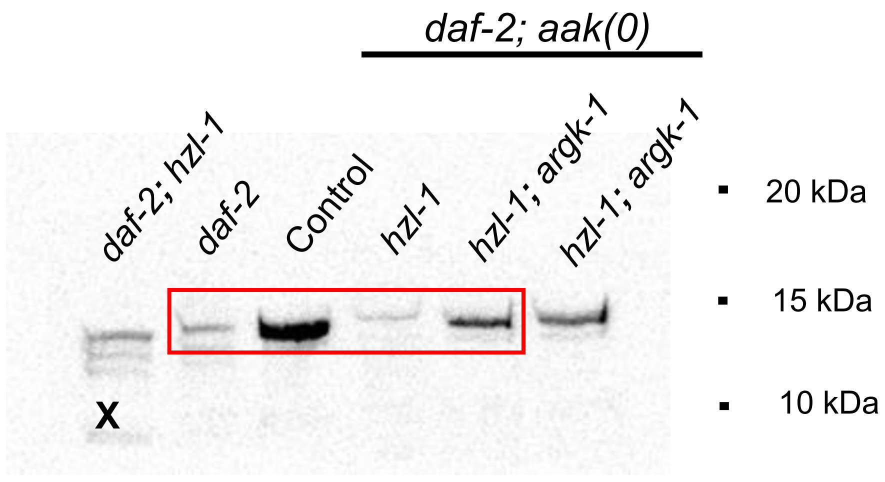

anti-h4EBP1

8% SDS-PAGE gel
Bottom half

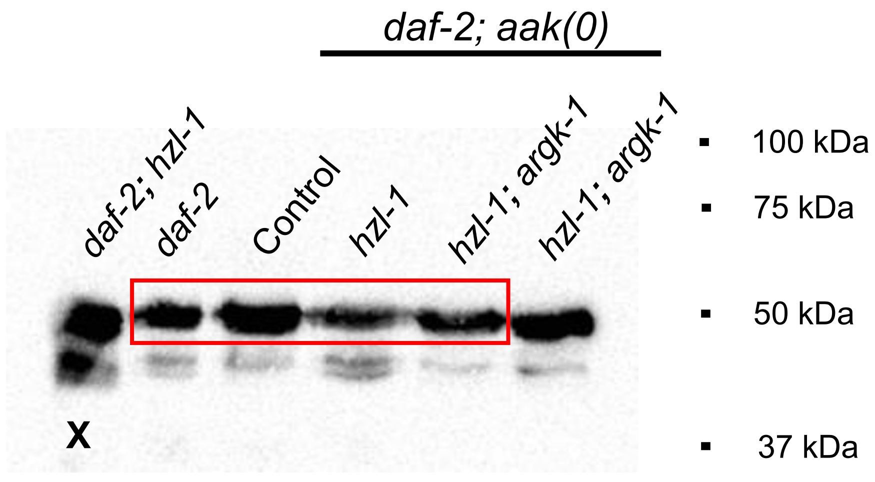

anti-α-Tubulin

8% SDS-PAGE gel
Top half

From Fig 6F) Levels of P-h4EBP1 used as a proxy for TOR activity detected by Western blot. Anti-P-h4EBP1 antibodies were used to detect phosphorylated h4EBP1 levels in animals. α-tubulin serves as a loading control. Dauer animals in the indicated genetic backgrounds were used as samples.

Western analyses were performed on Day 2 dauer larvae for each experiment. Approximately ~600 dauer larvae were used for each well, run on an 8% SDS-PAGE gel for 45 to 90 minutes, as needed. P-h4EBP1 and α-tubulin bands are from the same respective gels, with membranes cut and separated after membrane transfer for simultaneous antibody incubation.
