# Supplementary material for: AMPK modulates a DEAH box RNA-helicase to attenuate TOR signaling and establish developmental quiescence in Caenorhabditis elegans
Source: PLoS Biol. 2025 Dec 1;23(12):e3003144. doi: 10.1371/journal.pbio.3003144 (PMC12685192; doi:10.1371/journal.pbio.3003144)
Supplement: S1 Table — (PDF) [file pbio.3003144.s011.pdf]

**S1 Table. List of *C. elegans* strains used in study**

*All strains are from this study, unless indicated otherwise.

| Strain | Genotype | Source* |
|---|---|---|
| CB1370 | *daf-2(e1370)* | [1] |
| MR1000 | *daf-2(e1370); aak-1(tm1944) III; aak-2(ok524) X* | [2] |
| MR2511 | *mkcSi13 [sun-1p::rde-1::sun-1 3' UTR + unc-119(+)] II; daf-2(e1370); aak-1(tm1944) III; rde-1(mck36) V; aak-2(ok524) X* | [3] |
| MR2402 | *daf-2(e1370); aak-1(tm1944) III;  rde-1(mck36) V; aak-2(ok524) X; rrEx989 [sur-5p::rde-1::sun-1 3' UTR + unc-119(+)]* | |
| VC | *hzl-1(ok1688)* | CGC |
| MR2836 | *daf-2(e1370) III; hzl-1(ok1688) V* | |
| MR2837 | *daf-2(e1370) aak-1(tm1944) III; hzl-1(ok1688) V; aak-2(ok524) X* | |
| MR2378 | *daf-2(e1370) aak-1(tm1944) III; hzl-1(ok1688) V; aak-2(ok524) X ; rrEx778(hzl-1p::gfp::hzl-1)* | |
| MR2381 | *daf-2(e1370) aak-1(tm1944) III; hzl-1(ok1688) V; aak-2(ok524) X; rrEx781(hzl-1p::gfp::hzl-1)* | |
| MR2387 | *daf-2(e1370) aak-1(tm1944) III; hzl-1(ok1688) V; aak-2(ok524) X ; rrEx787(rgef-1p::gfp::hzl-1* | |
| MR2390 | *daf-2(e1370) aak-1(tm1944) III; hzl-1(ok1688) V; aak-2(ok524) X ; rrEx790(myo-3::gfp::hzl-1)* | |
| MR2393 | *daf-2(e1370) aak-1(tm1944) III; hzl-1(ok1688) V; aak-2(ok524) X ; rrEx793(wrt-2::gfp::hzl-1* | |
| MR2396 | *daf-2(e1370) aak-1(tm1944) III; hzl-1(ok1688) V; aak-2(ok524) X ; rrEx796(nhx-2p::gfp::hzl-1)* | |
| MR3101 | *daf-2(e1370) III; aak-1(tm1944)III; hzl-1(ok1688)V; aak-2(ok524) X; rrEx980(hzl-1p::GFP:hzl-1-S588D)* | |
| MR3102 | *daf-2(e1370) III; aak-1(tm1944)III; hzl-1(ok1688)V; aak-2(ok524) X; rrEx981(hzl-1p::GFP:hzl-1-S636D)* | |

| | | |
|---|---|---|
| MR3103 | *daf-2(e1370) III; aak-1(tm1944)III; hzl-1(ok1688)V; aak-2(ok524) X; rrEx982(hzl-1p::GFP:hzl-1-S588+636D)* | |
| MR3104 | *daf-2(e1370) III; hzl-1(ok1688)V; rrEx983(hzl-1p::GFP:hzl-1-S588A]* | |
| MR3105 | *daf-2(e1370) III; hzl-1(ok1688)V; rrEx984(hzl-1p::GFP:hzl-1-S636A]* | |
| MR3106 | *daf-2(e1370) III; hzl-1(ok1688)V; rrEx985(hzl-1p::GFP:hzl-1-S588A+636A]* | |
| MR3107 | *daf-2(e1370) III; aak-1(tm1944)III; hzl-1(ok1688)V; aak-2(ok524) X; rrEx986(nhx-2p::GFP:hzl-1-S588D)* | |
| MR3108 | *daf-2(e1370) III; aak-1(tm1944)III; hzl-1(ok1688)V; aak-2(ok524) X; rrEx987(nhx-2p::GFP:hzl-1-S636D)* | |
| MR3109 | *daf-2(e1370) III; aak-1(tm1944)III; hzl-1(ok1688)V; aak-2(ok524) X; rrEx988(nhx-2p::GFP:hzl-1-S588+636D)* | |
| MR2399 | *daf-2(e1370) aak-1(tm1944) III; hzl-1(ok1688) V; aak-2(ok524) X; rrEx799(nhx-2p::mKate::hzl-1ΔIDR1)* | |
| MR2400 | *daf-2(e1370) aak-1(tm1944) III; hzl-1(ok1688) V; aak-2(ok524) X; rrEx800(nhx-2p::mKate::HZLΔ1-IDR2)* | |
| MR2401 | *daf-2(e1370) aak-1(tm1944) III; hzl-1(ok1688) V; aak-2(ok524) X; rrEx801(nhx-2p::mKate::hzl-1ΔIDR3)* | |
| MR2786 | *daf-2(e1370) aak-1(tm1944) III; hzl-1(ok1688) V; aak-2(ok524) X; rrEx833(nhx-2p::mKate::hzl-1ΔIDR1+2)* | |
| MR2787 | *daf-2(e1370) aak-1(tm1944) III; hzl-1(ok1688) V; aak-2(ok524) X; rrEx834(nhx-2p::mKate::hzl-1ΔIDR2+3),* | |
| MR2788 | *daf-2(e1370) aak-1(tm1944) III; hzl-1(ok1688) V; aak-2(ok524) X; rrEx835(nhx-2p::mKate::hzl-1ΔIDR1+3)* | |
| MR2789 | *daf-2(e1370) aak-1(tm1944) III; hzl-1(ok1688) V; aak-2(ok524) X; rrEx836(nhx-2p::mKate::hzl-1Δ IDR1+2+3)* | |
| MR2774 | *daf-2(e1370) III; hzl-1(ok1688) V; rrEx796(nhx-2p::gfp::hzl-1)* | |
| MR2838 | *daf-2(e1370) aak-1(tm1944) III; aak-2(ok524) X; rrEx866(nhx-2p::gfp)* | |
| MAH205 | *argk-1(ok2993) V* | [4] |
| MR3080 | *daf-2(e1370) III; argk-1(ok2993) V* | |

| | | |
|---|---|---|
| MR3081 | *daf-2(e1370) aak-1(tm1944) III; argk-1(ok2993) V; aak-2(ok524) X* | |
| MR3082 | *daf-2(e1370) aak-1(tm1944) III; argk-1(ok2993) hzl-1(ok1688) V; aak-2(ok524) X* | |
| ST9013 | *ncEx9013 [lin-32p::FLAG::h4EBP1 + rol-6(su1006)]* | [5] |
| MR3094 | *daf-2(e1370) III; ncEx9013 [lin-32p::FLAG::h4EBP1 + rol-6(su1006)]* | |
| MR3095 | *daf-2(e1370); aak-1(tm1944) III; aak-2(ok524) X; ncEx9013 [lin-32p::FLAG::h4EBP1 + rol-6(su1006)]* | |
| MR3096 | *daf-2(e1370) aak-1(tm1944) III; hzl-1(ok1688) V; aak-2(ok524) X; ncEx9013 [lin-32p::FLAG::h4EBP1 + rol-6(su1006)]* | |
| MR3097 | *daf-2(e1370) aak-1(tm1944) III; argk-1(ok2993) hzl-1(ok1688) V; aak-2(ok524) X; ncEx9013 [lin-32p::FLAG::h4EBP1 + rol-6(su1006)]* | |
| DV3525 | *daf-15(re257[daf-15::mNG::AID]) IV* | [6] |
| CA1202 | *ieSi57 [eft-3p::TIR1::mRuby::unc-54 3'UTR + Cbr-unc-119(+)] II* | [7] |
| CA1472 | *ieSi68 [sun-1p::TIR1::mRuby::htp-1 3'UTR + Cbr-unc-119(+)] II* | [7] |
| MR3086 | *daf-2(e1370) III; ieSi68 [sun-1p::TIR1::MRuby::sun-1 3'UTR + Cbr-unc-119(+)] II; daf-15[re257(daf-15::mNG::AID]) V* | |
| MR3085 | *daf-2(e1370) III; isSi57[eft-3p::TIR1::MRuby::unc-54 3'UTR + Cbr-unc-119(+)] II; daf-15[re257(daf-15::mNG::AID V* | |
| MR3063 | *ieSi68[sun-1p::TIR1::MRuby::sun-1 3'UTR + Cbr-unc-119(+)] II; daf-2(e1370) aak-1(tm1944) III; daf-15[re257(daf-15::mNG::AID]) V; aak-2(ok524) X* | |
| MR3064 | *isSi57[eft-3p::TIR1::MRuby::unc-54 3'UTR + Cbr-unc-119(+)] II; daf-2(e1370) aak-1(tm1944) III; daf-15[re257(daf-15::mNG::AID)] IV; aak-2(ok524) X* | |
| MR3083 | *ieSi68[sun-1p::TIR1::MRuby::sun-1 3'UTR + Cbr-unc-119(+)] II; daf-2(e1370) aak-1(tm1944) III; daf-15[re257(daf-15::mNG::AID)] IV; hzl-1(ok1688) V; aak-2(ok524) X* | |
| MR3084 | *ieSi68[sun-1p::TIR1::MRuby::sun-1 3'UTR + Cbr-unc-119(+)] II; daf-2(e1370) aak-1(tm1944) III; daf-15[re257(daf-15::mNG::AID]) ]) argk-1(ok2993) hzl-1(ok1688) V; aak-2(ok524) X* | |

| | | |
|---|---|---|
| MR3140 | *daf-2(e1370) aak-1(tm1944) III; hzl-1(rr203[HA::mKate::hzl-1]) (rr219[HZL-1 S588D]) V; aak-2(ok524)X* | |
| MR3141 | *daf-2(e1370) III; hzl-1(rr220[HA::mKate::hzl-1]) (rr220[HZL-1S588A]) V* | |
| MR3157 | *daf-2(e1370) III; hzl-1(rr203[HA::mKate::hzl-1]) (rr221[HZL-1 S636A]) V* | |
| MR3158 | *daf-2(e1370) aak-1(tm1944) III; hzl-1(rr203[HA::mKate::hzl-1]) (rr222[HZL-1 S36D]) V; aak-2(ok524)X* | |
| MR3159 | *daf-2(e1370) aak-1(tm1944) III; hzl-1(rr203[HA::mKate::hzl-1]) (rr223[HZL-1 IDR1 del]) V; aak-2(ok524)X* | |
| MR3160 | *daf-2(e1370) aak-1(tm1944) III; hzl-1(rr203[HA::mKate::hzl-1]) (rr224[HZL-1 IDR3 del]) V; aak-2(ok524)X* | |
| MR3161 | *daf-2(e1370) aak-1(tm1944) III; hzl-1(rr203[HA::mKate::hzl-1]) (rr225[HZL-1 IDR2 del]) V; aak-2(ok524)X* | |
| MR3162 | *daf-2(e1370) aak-1(tm1944) III; hzl-1(rr203[HA::mKate::hzl-1]) (rr227[HZL-1-Heli del ]) V; aak-2(ok524)X* | |
| MR3163 | *daf-2(e1370) aak-1(tm1944) III; hzl-1(rr203[HA::mKate::hzl-1]) (rr226 [HZL-1 IDR 1+3 del]) V; aak-2(ok524)X* | |
| MR3100 | *daf-2 (e1270) III; argk-1p::argk-1::mCherry v* | |
| MR3167 | *daf-2(e1370) aak-1(tm1944)III; C44H9.4(ok1688) argk-1p::argk-1::mCherry V; aak-2(ok524)X* | |
| MR3166 | *daf-2(e1370) aak-1(tm1944)III; argk-1p::argk-1::mCherry V; aak-2(ok524)X* | |


1. Kimura KD, Tissenbaum HA, Liu Y, Ruvkun G. daf-2, an insulin receptor-like gene that regulates longevity and diapause in Caenorhabditis elegans. Science (New York, NY). 1997;277(5328):942-6.
2. Kadekar P, Roy R. AMPK regulates germline stem cell quiescence and integrity through an endogenous small RNA pathway. PLoS Biol. 2019;17(6):e3000309.

3. Zou L, Wu D, Zang X, Wang Z, Wu Z, Chen D. Construction of a germline-specific RNAi tool in C. elegans. Sci Rep. 2019;9(1):2354.
4. McQuary PR, Liao CY, Chang JT, Kumsta C, She X, Davis A, Chu CC, Gelino S, Gomez-Amaro RL, Petrascheck M, Brill LM, Ladiges WC, Kennedy BK, Hansen M. C. elegans S6K Mutants Require a Creatine-Kinase-like Effector for Lifespan Extension. Cell Rep. 2016 Mar 8;14(9):2059-2067. doi: 10.1016/j.celrep.2016.02.012. Epub 2016 Feb 25.
5. Nukazuka A, Tamaki S, Matsumoto K, Oda Y, Fujisawa H, Takagi S. A shift of the TOR adaptor from Rictor towards Raptor by semaphorin in C. elegans. Nat Commun. 2011 Sep 27;2:484. doi: 10.1038/ncomms1495.
6. Duong T, Rasmussen NR, Ballato E, Mote FS, Reiner DJ. The Rheb-TORC1 signaling axis functions as a developmental checkpoint. Development. 2020 Mar 2;147(5):dev181727. doi: 10.1242/dev.181727.
7. Zhang L, Ward JD, Cheng Z, Dernburg AF. The auxin-inducible degradation (AID) system enables versatile conditional protein depletion in C. elegans. Development. 2015 Dec 15;142(24):4374-84. doi: 10.1242/dev.129635. Epub 2015 Nov 9.
